# Potential of CDC25 phosphatases in cancer research and treatment: key to precision medicine

**DOI:** 10.3389/fphar.2024.1324001

**Published:** 2024-01-19

**Authors:** Ibraheem Dakilah, Amani Harb, Eman Abu-Gharbieh, Waseem El-Huneidi, Jalal Taneera, Rifat Hamoudi, Mohammed H. Semreen, Yasser Bustanji

**Affiliations:** ^1^ Research Institute of Medical and Health Sciences, University of Sharjah, Sharjah, United Arab Emirates; ^2^ Department of Basic Sciences, Faculty of Arts and Sciences, Al-Ahliyya Amman University, Amman, Jordan; ^3^ College of Medicine, University of Sharjah, Sharjah, United Arab Emirates; ^4^ Division of Surgery and Interventional Science, University College London, London, United Kingdom; ^5^ College of Pharmacy, University of Sharjah, Sharjah, United Arab Emirates; ^6^ School of Pharmacy, The University of Jordan, Amman, Jordan

**Keywords:** cancer, CDC25, natural compounds, AI (artificial intelligence), precision medicine, omics

## Abstract

The global burden of cancer continues to rise, underscoring the urgency of developing more effective and precisely targeted therapies. This comprehensive review explores the confluence of precision medicine and CDC25 phosphatases in the context of cancer research. Precision medicine, alternatively referred to as customized medicine, aims to customize medical interventions by taking into account the genetic, genomic, and epigenetic characteristics of individual patients. The identification of particular genetic and molecular drivers driving cancer helps both diagnostic accuracy and treatment selection. Precision medicine utilizes sophisticated technology such as genome sequencing and bioinformatics to elucidate genetic differences that underlie the proliferation of cancer cells, hence facilitating the development of customized therapeutic interventions. CDC25 phosphatases, which play a crucial role in governing the progression of the cell cycle, have garnered significant attention as potential targets for cancer treatment. The dysregulation of CDC25 is a characteristic feature observed in various types of malignancies, hence classifying them as proto-oncogenes. The proteins in question, which operate as phosphatases, play a role in the activation of Cyclin-dependent kinases (CDKs), so promoting the advancement of the cell cycle. CDC25 inhibitors demonstrate potential as therapeutic drugs for cancer treatment by specifically blocking the activity of CDKs and modulating the cell cycle in malignant cells. In brief, precision medicine presents a potentially fruitful option for augmenting cancer research, diagnosis, and treatment, with an emphasis on individualized care predicated upon patients’ genetic and molecular profiles. The review highlights the significance of CDC25 phosphatases in the advancement of cancer and identifies them as promising candidates for therapeutic intervention. This statement underscores the significance of doing thorough molecular profiling in order to uncover the complex molecular characteristics of cancer cells.

## 1 Introduction

Cancer is the unregulated and rampant replication of cells that leads to the disease one can witness in all population groups. Cancer diagnoses have been steadily rising in the younger demographic worldwide (Ugai et al., 2022) and the search for more effective and targeted therapies continues. Some of the deadliest of these reported globally were breast cancer, stomach cancer, non-melanoma skin cancer, colon and rectum cancer, cancers affecting the respiratory system/tract, and prostate cancer have increased (Lin et al., 2021; Semreen et al., 2022; Ahmed et al., 2023; Hagyousif et al., 2023). Unfortunately, the progress of ready-to-market therapeutics has not increased at the same rate with many of the drugs still in clinical trials due to confounding data, poor clinical trial management and experimentation on top of the inherent duration of sufficient clinical experimentation for such novel treatments (Schilsky, 2010). Novel treatments are sought for their effectiveness against current treatment-resistant and aggressive tumours, increasing the prognosis for cancer patients (Motawi et al., 2014; Alfarouk et al., 2015). Another avenue that has in recent years been making strides in delivering a more personalized and purported more effective treatment has been the field of precision medicine. Precision medicine considers the pharmacological and genomic effects that arise from person to person delivering an effective treatment on a case-by-case basis.

## 2 Definition and significance of precision medicine

Precision medicine, also known as personalized medicine, is a healthcare technique that incorporates each patient’s distinctive characteristics when making decisions regarding their medical treatment. It reflects that people differ in terms of their genetic profile, environmental exposures, lifestyle choices, and illness features. Precision medicine, instead of a one-size-fits-all strategy, tries to personalize medical therapies to each patient’s needs (Tsimberidou et al., 2020). Founded on the recognition that individuals may respond differently to therapies depending on the previously mentioned profiles. Precision medicine tries to find particular biomarkers or genetic abnormalities linked with certain diseases, such as cancer, by applying modern technologies involving combinations of genomic sequencing, molecular profiling, and bioinformatics (Olivier et al., 2019). This helps healthcare providers forecast an individual’s likelihood of acquiring particular disorders and select the most effective treatment choices. Observations from many cancers cases have shown to be unresponsive to traditional chemotherapy, the reasons which can be found in the patient tumour molecular profile.

Precision medicine is critical in cancer research due to how it improves diagnostic accuracy, optimizes treatment choices, and improves patient outcomes. Precision medicine can uncover specific genetic variations or modifications fuelling the growth of cancer cells by examining a patient’s tumour at the molecular level (Roelands et al., 2023). This knowledge can help lead to the development of tailored medicines that directly target these molecular anomalies, boosting the likelihood of a positive response (Tsimberidou et al., 2020). More accurate methods of screening arising from advancements in algorithmic imaging and sorting and learning from data sets have reduced extraneous harm as fewer patients would need to undergo radiotherapy (Avanzo et al., 2020). Furthermore, precision medicine promotes the development of biomarker-driven clinical trials, which aim to test the efficacy of new medicines on patient subgroups with similar genetic or molecular traits. This approach enables researchers to find novel therapeutic targets and hasten the development of innovative medications that are more effective and less hazardous (Abushawish et al., 2022; Ahmed et al., 2023).

## 3 Introduction to CDC25 phosphatases and their role in cell cycle regulation

These new treatments attempt to tackle the mechanism by which cell replication or innate repair goes awry leading to tumorigenesis. One promising avenue of research is the use of inhibitors of cell cycle division. Cycle Division Cycle 25 (CDC25), a CDK phosphatase (Lavecchia et al., 2009), plays a vital role in regulating the factors of cyclic expression observed for cell cycle progression making it an attractive target for targeted cancer therapeutics. As such, CDC25 phosphatases are some of the more attractive targets for cancer therapy, especially for cancer types that are more aggressive and more difficult to treat such as receptor protein triple-negative breast cancer (He et al., 2013; Liu et al., 2018).

CDC25 proteins are phosphatases expressed during the cell cycle regulating factors including CDKs (Hoffmann and Karsenti, 1994). CDKs are crucial for the progression and regulation of the interphase and entry into mitosis. The regulation of CDKs occurs through the phosphatase action of the three different paralogues of CDC25. The well-documented component of CDC25 isoforms is CDC25A expressed and active during G1/S and G2/M checkpoints, while CDC25B and CDC25C are active during the G2/M checkpoints and have additional roles, including DNA damage repair and regulation of meiosis respectively.

In this review, we will discuss the actions of CDC25 phosphatases in causing neoplastic growth and cell cycle regulation, the overview of CDC25 inhibitors and efficacy in different cancers and the development of CDC25 inhibitors for cancer therapy both *in-vitro* and clinical trials, and finally review the complications and future for human cancer treatments.

## 4 Exploring the link between precision medicine, CDC25 phosphatases, and the potential of CDC25 inhibitors in cancer treatment

**FIGURE 1:**
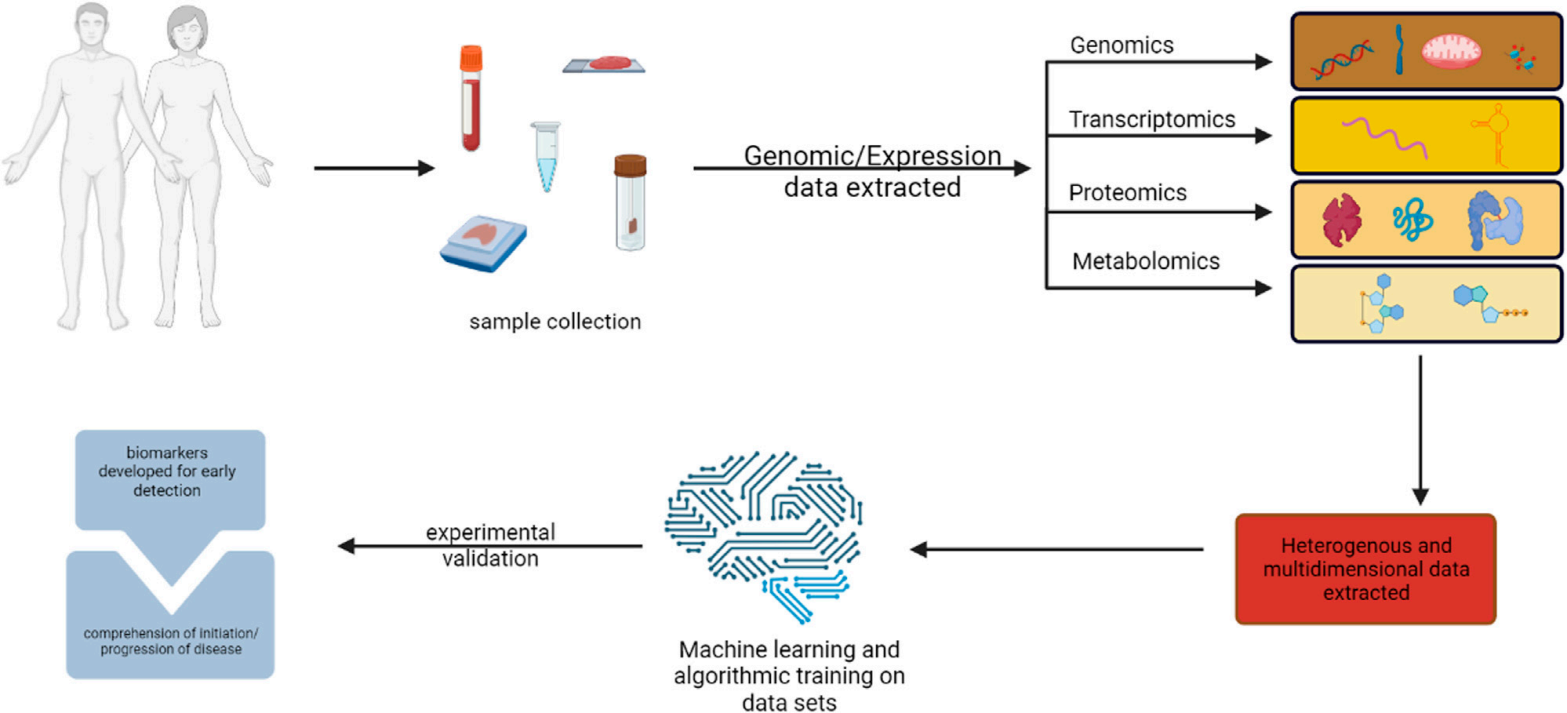
illustration of multi-omics methods for finding cancer biomarkers for early diagnosis and pathogenesis. Created with BioRender.com.

The main targets of precision medicine and *in silico* techniques are the molecular or metabolomic profiles that can be used to accurately predict patients’ risk for cancer and detect cancerous tissue at an early stage (Li et al., 2022). Clinical sequencing investigations have established that genomic profiling is feasible in clinical settings and that it is possible to build procedures for informing patients and healthcare professionals about the results of these studies (Forrest et al., 2018). Omics’ technique matching scores were associated with better disease control rates, suggesting that customizing combination therapies based on individual genomic alterations may lead to improved outcomes (Figure 1). High matching rates were found, primarily due to comprehensive molecular profiling, timely Molecular Tumour Board discussions, and rapid access to drugs (Sicklick et al., 2019; Dahabiyeh et al., 2022; Semreen et al., 2022; Dahabiyeh et al., 2023). Screenings that preceded cancer therapies to tailor therapy are in clinical trials, for instance, endometrial cancer falls that have distinct molecular profiles are actively being investigated with antagonists or inhibitors that are pathological molecular markers. Markers in this particular study play very similar roles in activating cell cycle progression to CDC25 as PTEN, PI3K pathway activate Cyclin D1 (Arend et al., 2018).

The potential for CDC25 as a therapeutic target arising from a precise analysis of various tumours can be related to cell cycle markers found in previous studies and their results. Molecular markers that are putatively ubiquitous with some cancers may not be directly related to the cell cycle, as in they do not directly act on the Cyclins, Cdks or checkpoint proteins involved, but they may affect the proliferation of cells or indicate an irregularity in cell cycle regulation as the case is for PD-L1 in various cancers (Schulz et al., 2021) even those with more complex ontology (Banchereau et al., 2021). Precision medicine studies involving machine learning algorithms were able to decipher from complex and heterogenous genomic (Tang et al., 2023) data the potential efficacy of using the PD-L1 inhibitors on various cancers based on molecular determinants of the tumours via transcriptional profiling using RNA sequencing under immune checkpoint inhibitors (Banchereau et al., 2021; Chen et al., 2021). Results from this can be used to inform and direct treatment-responsive patients to those respective therapeutic agents (Cristescu et al., 2018) or radiotherapeutic procedures (He et al., 2020; Johannet et al., 2021). The checkpoint PD-1 and PD-L1, similar to CDC25 have been observed to sustain long term remission by utilizing personalized and genomic based targeting specifically in melanoma (Axelrod et al., 2018; Petrova et al., 2020).

An advantage of some of the aforementioned conjugation of *in silico* and *in vitro* methods mentioned above is that new patterns and markers can be retrieved from previously archived data that may be obscured by the noise of redundant pathways (Salameh et al., 2022). The image analysis of the microarrays, histographs and tumour sections are closer to being linked than previously thought using these machine learning methods to link the pathophysiological characteristics of those objects to the molecular profiles (Chua et al., 2021). Trials that used biomolecular markers such as epidermal growth factor receptors targeted after genomic analysis of patients’ tumor profiles’, observing a significant result without chemotherapy using immunotherapy (Hainsworth et al., 2018). In corollary, these same methods can be used to analyze drug libraries to ascertain whether chemical structures and properties of archival drugs can be repurposed for novel chemotherapeutic purposes (Issa et al., 2021; Tao et al., 2021) and with the expansion of click chemical construction many more can be constructed using the existing functional backbones to overcome resistance (Tao et al., 2021; Vartak et al., 2023; Chowdhary et al., 2024), which leaves hope that there may already be an anti-CDC25 agent or a precursor that is awaiting further testing or reconfiguration.

## 5 Cell cycle regulation and CDC25 phosphatases

### 5.1 Overview of the cell cycle and its importance in cellular function

**FIGURE 2:**
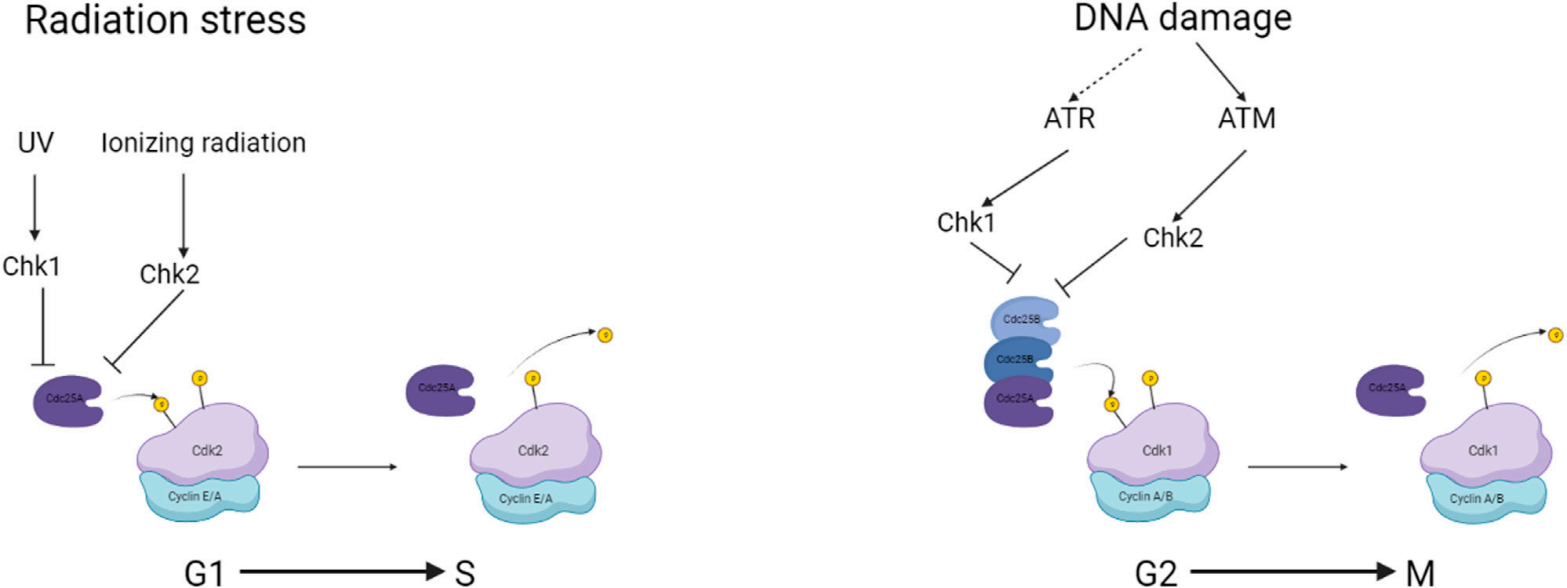
a simplified overview of checkpoints associated with CDC25 and the regulation during genomic damage. Created with BioRender.com.

The cell cycle in somatic cells is divided into two main parts, interphase in which the cell replicates DNA, growth factors and proteins required for the subsequent mitotic division to create the two daughter cells. Some cells enter the cell cycle from a G0 or gap phase after interacting with a mitogenic factor. The cells will then enter G1 followed by a phase specifically for replicating DNA, an S phase a G2 phase where checks on the integrity of the replicated DNA occur and finally the cell’s mitotic M phase and cytokinetic division. Between each phase, there are figurative checkpoints that are regulated by the activation and complexing of CDKs with Cyclins (Figure 2). The regulation and control of cells determined to enter the cell cycle begin at the G1 phase with the interaction of Cyclin D with CDK4/6. This CDK-Cyclin complex interacts with Retinoblastoma protein (RB) releasing chromatin remodelling enzymes and transcription factors triggering the expression of downstream CDK. The activation of these signals and regulating proteins such as RB is achieved through the phosphorylation of amino acid residues. CDK-Cyclin complexes are both activated and inactivated by similar mechanisms. The activity of the complexes is repressed mainly by the phosphorylation of Threonine14 and Tyrosine15 catalyzed by WEE1 and MYT1 kinases (Agius et al., 2015). The activation required for cell cycle progression is completed by the protein phosphatase CDC25.

### 5.2 Explanation of CDC25 phosphatases and their role in cell cycle progression

As with many of the mitotic factors and cell cycle regulation control proteins, CDC25 was first discovered in the fission yeast *Schizosaccharomyces pombe* (Hoffmann and Karsenti, 1994) as a positive regulator of CDC2 now known as CDK1 (Russell and Nurse, 1986). CDK1 activation ensures that cells within interphase can commit to mitotic division (Bretones et al., 2015). As mentioned before the role of CDC25 as a protein phosphatase is to remove the inhibitory phosphate group from Tyr15 of CDKs involved in progression through the cell cycle checkpoints. Activation of CDK through binding with Cyclins and potential phosphorylation by protein tyrosine kinases (PTK) leads to activation and progression through the cell cycle leading to mitotic entry and continuation of the cell cycle. The constitutive activation of CDK-Cyclin complexes has been implicated in the initiation of many cancers, with constitutively active CDK-Cyclin complexes ensuring continuous kinase activity and activation of cell cycle factors such as downstream CDKs and chromatin remodelling enzymes. Leading to an aberrant cell cycle and unregulated cell division, forming a neoplasm that may progress into a cancerous cell mass if left unchecked.

CDC25 not only has an effect on the activation of the CDK-Cyclin complex but another regulator of cell cycles, the Raf/MEK/ERK pathway. In prostate cancers, CDC25A inhibitors were used and found to inhibit the downstream activation of MEK with CDC25A directly (Nemoto et al., 2004). The Raf/MEK/ERK pathway is activated by growth factors interacting with growth factor receptors resulting in the downstream activators of transcription factors such as AP-1, an important trans-acting factor involved in regulating the expression of cell proliferation, differentiation, and apoptosis. CDC25A was hypothesized to remove the inhibitory phosphate group from Raf as seen with the hyperphosphorylation of Raf after the minimal application of CDC25 inhibitor, NSC 95397 or NSC 672121 (Nemoto et al., 2004). Specifically, a putative proto-oncogene that has been more directly implicated in cancer and connected to the Raf/MEK/ERK pathway is the transcription factor c-Myc, inducing DNA replication by binding to activator sites (Bretones et al., 2015).

The ability of CDC25 inhibitors to cause cell cycle arrest and death in cancer cells makes them promise as cancer treatment agents. This is accomplished by inhibiting CDK activation, which inhibits cell division and regulates the cell cycle. Because normal cells are less dependent on CDC25 for cell division and cell cycle control than cancer cells, the mechanism of action of CDC25 inhibitors renders them less harmful to them.

### 5.3 Dysregulation of CDC25 phosphatases in cancer and its impact on tumour formation

Dysregulation and constitutive expression of CDC25 have shown to be a constitutional mechanism in some cancers, with an overexpression observed and implicated in clinical outcomes of breast, ovarian and colorectal cancer patients (Figure 2). In the case of ovarian cancer studies, found that the overexpression investigated using immunohistochemistry that the poor prognosis had a link to the overexpression of CDC25A and CDC25B in a sample of 106 patients (Broggini et al., 2000). Additionally, breast cancer resistant to ionizing radiation was found to overexpress CDC25A (Löffler et al., 2003). CDC25A and CDC25B showed strong correlations to high-grade lymphoma reported as aggressive (Kristjánsdóttir and Rudolph, 2004). Cdc25A and Cdc25B were also overexpressed in non-Hodgkin’s lymphoma and various other cancers, including oesophageal, gastric, lung, thyroid, and head and neck cancers (Kristjánsdóttir and Rudolph, 2004). Exclusive overexpression of CDC25A in hepatocellular carcinoma is rare (Xu et al., 2003), while pancreatic ductal carcinoma and gastric carcinomas exclusively overexpress Cdc25B (Kristjánsdóttir and Rudolph, 2004).

All three isoforms of CDC25 would be considered proto-oncogenes, with the overexpression or constitutive activation leading to premature entries into either the S or M phase of the cell cycle. The role of Myc protein in overexpressing CDC25 is rather complicated and according to the literature quite contested. It appears that the role of Myc in overexpressing CDC25 follows cancer-specific patterns. In lymphoma and certain lung cancers, there was a clear correlation between the overexpression of Myc and CDC25 (Hernández et al., 1998). Some speculate that the interaction and overexpression of CDC25 in relation to MYC is context-dependent, as both CDC25A and CDC25B proteins have Myc target sites (Galaktionov et al., 1996; Kristjánsdóttir and Rudolph, 2004).

A big discovery was the fact that CDC25 overexpression did not drive cell proliferation with an absence of correlation found between the expression and rate of proliferation in many studies (Hernández et al., 1998; Cangi et al., 2000; Miyata et al., 2000). This is most likely due to the involvement of other necessary growth factors and signalling cascades required for the expression of cell cycle genes. It does appear that CDC25 overexpression allows for bypassing checkpoints involved in checking for genomic damage before entering S and M phases (Kristjánsdóttir and Rudolph, 2004).

## 6 Precision medicine and molecular profiling

### 6.1 Definition and purpose of precision medicine in cancer treatment

Precision medicine is a cutting-edge medical strategy that examines the genetic, genomic, epigenetic, and proteomic changes present in cancer cells using cutting-edge molecular profiling technology. Precision medicine enables oncologists to choose tailored therapies that directly interfere with the aberrant signalling pathways responsible for tumor genesis and progression by discovering particular biological drivers of cancer growth and survival (Naithani et al., 2021). Precision medicine therapies are intended to specifically attack cancer cells while preserving healthy organs, hence lowering treatment-related adverse effects, in contrast to standard chemotherapy, which broadly targets rapidly dividing cells.

Personalized methods are being made critical to cancer research due to improvements in diagnostic accuracy, optimizing treatment choices, and generally improving patient outcomes. Precision medicine can uncover specific genetic variations or modifications fuelling the growth of cancer cells by examining a patient’s tumour at the molecular level. This knowledge can help lead to the development of tailored medicines such as individual immunotherapy that directly target personalized and distinct immunophenotypes (Zeggini et al., 2019) that can be formulated from the previously mentioned molecular aberrations, boosting the likelihood of a positive response (Naithani et al., 2021). Furthermore, precision medicine promotes the development of biomarker-driven clinical trials, which aim to test the efficacy of new medicines on patient subgroups with similar genetic or molecular traits (Kong et al., 2022). This approach enables researchers to find novel therapeutic targets and hasten the development of innovative medications that are more effective and less hazardous.

With the explosion of machine learning models and AI-driven methods for pattern recognition in large biological data sets it appears that precision medicine may only become more granular and effective by detecting the relevant and appropriate markers associated with detection and for relating patients to the correlating effective treatment (Bhinder et al., 2021; Nayarisseri et al., 2021). In addition to molecular identification of personal biomarkers, archival images retrieved from radiotherapy can be used to glean the efficacy of other radiological treatments for personal cancers (Viswanathan et al., 2014). By incorporating radiomics into cancer research, we may combine imaging data with other genetic profiling methods to better understand the heterogeneity of the tumour and develop individualized therapy plans encompassing post-procedure lifestyle and care (Wang et al., 2022). As it provides a non-invasive and therapeutically accessible way to evaluate the genetic properties of tumours and track treatment outcomes over time, this technology is crucial to the field of precision medicine.

### 6.2 Introduction to molecular profiling techniques for identifying molecular alterations in cancer cells

**FIGURE 3:**
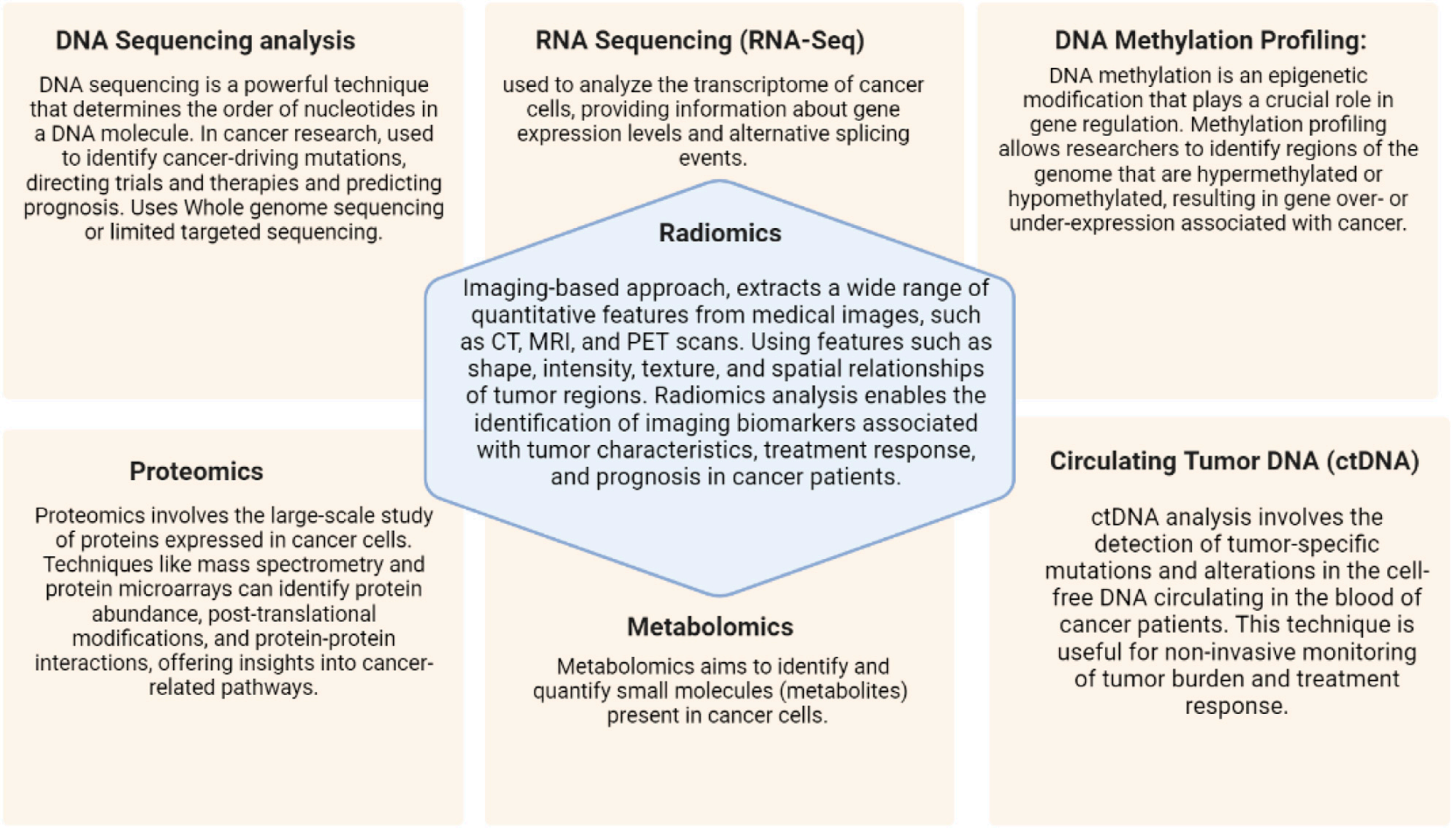
Molecular profiling techniques and imagining in precision oncology.

Cancer is a challenging and heterogeneous pathology that is triggered by genetic and molecular changes that impair regular cellular functions and cause unchecked cell growth and proliferation. For the purpose of improving cancer research, diagnostic, and treatment methods, it is crucial to comprehend the molecular environment of cancer cells. The development of comprehensive molecular profiling tools has advanced significantly over the past two decades, revolutionizing our ability to identify the genomic, transcriptomic, epigenomic, and proteomic changes found in cancer cells (Figure 3). Using previous data points precision medicine has also been able to identify radiomic response of certain molecular mutations inherent in some populations of cancer, easing both the load on patient and healthcare providers (Rosario et al., 2018).

Liquid biopsies, such as circulating tumor DNA and circulating tumor cells, present promising results for non-invasive tools for detecting and monitoring Small cell lung carcinoma, providing insights into tumor heterogeneity and potential therapeutic targets (Meijer et al., 2022). Non-invasive methods in combination with personalized medical techniques, increasing turnaround times and detection for particularly heterogeneous cancer types such as colorectal cancer and prostate cancer (Vandekerkhove et al., 2019; Malla et al., 2022). Along with genomic and deep learning analyses biomolecular markers, potentially CDC25, can be used as predictive measures for predictive biomarkers for treatment outcomes and recurrence risk in cancers, emphasizing the need for further multicentred investigations (Nakano et al., 2023). Heterogeneity in major cancers such as breast and gastric cancers poses a major hurdle for therapy, with precision medicine it is proposed that it will be significantly easier to choose patients for certain treatments (Garrido-Castro et al., 2019; Wang et al., 2019). CDC25 can be identified from single-cell analysis due to cytoplasmic localization of the phosphatase in rapidly proliferating and continual cells (Gabrielli et al., 1996).

Other Omics technologies other than radiomics have utilities for either the identification of targets for developing and formulating small molecule inhibitors. Analysing proteomic alterations in protein expression or post-translational modifications throughout checkpoint inhibitor therapy facilitates the assessment of therapeutic efficacy and prognostication of patient outcomes. Proteomic scrutiny alongside learning platforms enables thorough and dynamic monitoring of shifts in protein profiles across different graded cancers (Mehlich and Marusiak, 2022; Monsivais et al., 2023), providing critical insights into treatment responsiveness and the emergence of resistance mechanisms (Yu et al., 2023). With the repository of microarray data, comparative and exploratory algorithms checking for overexpression between cancerous and benign tissue can illuminate subjects for treatment, such as checkpoint inhibitors. A similar basis can be used for CDC25 as was used for MCM6 (mini-chromosome maintenance), a vital protein for chromosome stability. Proteomics has linked other members within this protein family, finding overexpression between cancerous and normal tissue, particularly in bowel cancer (Wang et al., 2023). The utility of proteomics was not just singled out to this check-point inhibitor, Proteolysis targeting chimera D6 in triple-negative breast cancer along a CDC25-CDK1 axis was found by comparing triple-negative breast cancer cell line response against D6 (Wu Y. et al., 2024).

Later on in this review, we will expand on the subject of quinones, a derivative of which 6-isomer of 5,8-quinolinedione performed cytotoxically well in colorectal cell lines. A combination of proteomic and phosphorylation profiling was able to demonstrate the efficacy (Narwanti et al., 2023), presenting other avenues for trialling small molecule inhibitors. Within the precision medicine parameter integration of proteomics, particularly concerning small molecule inhibitors, empowers clinicians and researchers with intricate molecular insights. Through mass spectrometry-based techniques, protein expression levels, post-translational modifications, and interactions are comprehensively analyzed. Using machine learning models and evolving functional and spatial mathematical modelling reveals cellular pathways that are vital for stemminess a crucial property of understanding cancer therapeutics (Plattner et al., 2023). This synthesis aids in the tailored selection, validation, and optimization of therapeutic strategies, advancing the realization of precision medicine’s promise for personalized and effective patient care (He and Wang, 2023; Plattner et al., 2023; Srivastava et al., 2024).

Other revolutionary technological advance representing a synergy of large cancer data analysis and diagnosis/discovery is metabolomics, using low molecular weight metabolites produced by various aberrant cellular processes (Carapito et al., 2024). Underpinned by the reality that cancer cells have different metabolic profiles than normal cells and can be used to find biomarker signature. Glutamine, a pivotal metabolite, acts as a versatile substrate intricately involved in a myriad of biosynthetic pathways critical for the unchecked proliferation of cancer cells. Serving as a fundamental building block fuelling diverse cellular processes, encompassing the synthesis of nucleotides, amino acids, and fatty acids essential for sustaining the rapid growth and expansion characteristic of malignant cells making the catabolic enzyme glutaminase a valuable target for cancer therapeutics (Cyriac and Lee, 2024). Other profiles that are used for research purposes and hopefully another facet for therapeutic discovery is the lipid profile (Wu Q. et al., 2024) and kynurenine resulting from liver metabolism, demonstrating results with a high precision and specificity using machine learning models (Wu Q. et al., 2024) illuminating other potential targets for cancer treatment or diagnosis by finding enzymes and differentially regulated genes within their respective pathways (Dai et al., 2023). Another subset of metabolomics, volatile organic compound–omics presents another potentially valuable avenue of research using the volatile profile of human exudates or excretions in, for example, bladder cancer marker identification (Carapito et al., 2024).

The traditional molecular techniques used in conjunction with the recent machine learning approaches have broadened the knowledge gap in some of the more difficult and more ontologically convoluted cancers such as prostate cancer. Most notably deep neural networks, a class of machine learning algorithms designed to model complex patterns and relationships in data by using multiple layers of interconnected artificial neurons, can predict Gleason scores from visuals of prostate cancers taken during histopathology, deep neural networks have so far produced excellent results (Nagpal et al., 2020).

## 7 Genetic variations in CDC25 phosphatases

As mentioned above there are three major isoforms of CDC25: CDC25A, CDC25B and CDC25C. The molecular weights of the three CDC25 isoforms range from 53 to 65 kDa. CDC25A and CDC25B comprise 524 and 580 amino acids respectively whilst CDC25C consists of only 473 (Brenner et al., 2014). The CDC25 protein structure is separated into two major regions: the N-terminal area and the C-terminal region. The N-terminal region is quite varied, including phosphorylation and ubiquitination sites governing phosphatase activity. The catalytic site is located at the C-terminus, which as mentioned before is quite consistent between the isoforms (Sur and Agrawal, 2016). The CDC25 family’s highly conserved area with uncertain function is the adjustable cysteine residue, Cys484, which is situated in a cleft binding to a sulphate group (Reynolds et al., 1999). Oxidation of active site cysteine has been suggested to be a part of a checkpoint for increasing the oxidation state within the cell, ROS attacking the cysteine leads to a triggering of this checkpoint (Kristjánsdóttir and Rudolph, 2004). Hotspots, considered essential for substrate identification, are situated around 20–30Å away from the active site (Sohn et al., 2004). All CDC25 isomers contain conserved catalytic domains but very different regulatory regions. Regulatory areas are subjected to alternative splicing events, which result in two variants for CDC25A and five each for CDC25B and CDC25C (Baldin et al., 1997; Wegener et al., 2000). The phosphatases’ intracellular location and turnover are determined by the non-catalytic domain. The general structure outlined has strong similarities to other dual specificity phosphatases involved in cellular communication and cell cycle regulation, which will have implications for inhibitor generation.

CDC25A is activated during the G1 phase of the cell cycle and is responsible for driving the cell through the G1 checkpoint and into the S phase. It is degraded by proteolysis via the ubiquitination mechanism at the end of mitosis (Donzelli et al., 2002). CDC25B is expressed during the G2 phase of the cell cycle and stimulates CDK1, which is necessary for mitosis to begin (Gabrielli et al., 1996). CDC25C is similarly involved in the mitotic entrance, but it is controlled by the checkpoint kinase CHK1 and is activated only after DNA damage has been repaired (Frazer and Young, 2012).

CDC25 activity is closely controlled by a variety of processes, including phosphorylation, proteolysis, and gene expression regulation. Checkpoint circuits that respond to DNA damage and other stimuli to limit cell cycle advancement until the damage is repaired also control CDC25 activity. Phosphorylation is one of the essential CDC25 regulatory mechanisms. A variety of kinases, including CHK1, CHK2, and ATM, phosphorylate CDC25 (Sur and Agrawal, 2016). CDC25 phosphorylation can either promote or inhibit its action, depending on the location phosphorylated and the kinase involved. For example, phosphorylation of CDC25 in response to DNA damage by CHK1 or CHK2 limits its function, preventing the cell from starting mitosis until the damage is repaired (Sur and Agrawal, 2016).

Proteolysis regulates CDC25 as well. After mitosis, the anaphase-promoting complex/cyclosome (APC/C) targets CDC25B for degradation, preventing it from activating CDK1 and sending the cell back into the G1 phase. Similarly, during the S phase, the SCFβ-TrCP complex targets CDC25A for degradation, preventing premature entrance into mitosis. Degradation is achieved by the phosphorylation of three serine residues by CHK1/CHK2 (Boutros et al., 2006). The MAPK5 pathway, JNK and p38 pathways, and checkpoint kinase Chk1 all target CDC25 phosphatases, particularly CDC25B, for degradation or inhibition, leading to cell cycle arrest or delay. CDC14A phosphatase can also inhibit the catalytic activity of CDC25B by dephosphorylating it, preventing premature entry into mitosis (Sur and Agrawal, 2016).

The final member of the CDC25 cohort, CDC25C, is inactivated by various protein kinases and phosphatases. Chk1, Cds1/Chk2, c-TAK1 kinase, and JNK are involved in the phosphorylation of CDC25C at Ser287, Ser168, and Thr138 (Liu et al., 2020), leading to the inactivation of CDC25C (Boutros et al., 2006; Sur and Agrawal, 2016). Phosphatases like PP1, PP2A, and hCDC14B dephosphorylate CDC25C to activate it, while the PP2A-B56δ complex negatively regulates CDC25C activity by dephosphorylating Thr138, leading to its exit from mitosis (Sur and Agrawal, 2016).

CDC25 expression is also controlled at the transcriptional level. The E2F family of transcription factors, which are activated by the RB protein, can promote cell cycle advancement by inducing the expression of CDC25A and CDC25B (Vigo et al., 1999; Sur and Agrawal, 2016). In contrast, the tumour suppressor protein p53 can limit CDC25C expression, preventing cells from entering mitosis in response to DNA damage.

## 8 CDC25 inhibitors in precision cancer medicine

### 8.1 Overview of CDC25 inhibitors and their classification

Many CDC25 inhibitors are currently being investigated for clinical use against tumorigenesis or the metastasis of more developed cancers. The initial testing as with all treatments starts with an *in-vitro* trial on the relevant cancer cell lines or tissue, and if proven to have successful inhibitory effects on cancer would then follow onto *in vivo* using graft models of humanized mice and then the clinical trials (Liu et al., 2020). However, this poses a challenge with CDC25 as there are 3 forms of the protein with slightly different structures and it has been shown that suppression of one may suppress the tumour growth but may not be sufficiently powerful to move forward into clinical testing and development of a ready-for-market treatment. The isoform predicament can also be seen as a blessing in disguise as some research shows that all three interactions are required for entry into M phase (Sur and Agrawal, 2016). In this section of the review, the origins, effects, and results of several putative cdc25 inhibitors will be summarized (Table 1). The three general types of CDC25 cancer inhibitors include small molecule inhibitors, peptide-based inhibitors, and natural product inhibitors.

**TABLE 1:** Mode of action and mechanism of potential anti-cancer compounds with the limitations/hurdles with the application for treatment. Progress in trials was either inferred from research papers or direct results from ClinicalTrials.gov.

Name of Cdc25 inhibitors	General inhibitor type	Molecule type	Mode of action	Sources
NSC-663284	Small molecule inhibitor	Quinolinedione	Blocking the binding of CDC25A	Lazo et al. (2002)
IRC-083864	Small molecule inhibitor	Bis quinone	Binding to CDC25B	Brezak et al. (2009), Sarkis et al. (2017)
NSC-95397	Small molecule inhibitor	Quinone-based	Binding to all isoforms	Peyregne et al. (2005)
BN82685	Small molecule inhibitor	Quinone-based	Direct binding to CDC25	Brezak et al. (2009)
SN-38	Indirect inhibitor	active metabolite of irinotecan	Activation of CHK1/2	Ditano et al. (2021)
2-fluoro-4-hydroxybenzonitrile	Small molecule inhibitor	nitrile derivative	CDC25B catalytic domain binding	Lund et al. (2015)
Genistein	natural product, an indirect inhibitor	isoflavone compound (legume derived)	Activation of CHK1/2	Brenner et al. (2014)
Bozitinib	small molecule inhibitor	Amino pyrimidines	Inhibits activation of CDC25	Lavecchia et al. (2010)
UPD-140	Small molecule inhibitor	Naphthoquinone	Inhibits CDC25A	Kabakci et al. (2019)
UPD-176	Small molecule inhibitor	Naphthoquinone	Inhibits CDC25A	Kabakci et al. (2019)
UPD-172	Small molecule inhibitor	Naphthoquinone	Inhibits CDC25A	Kabakci et al. (2019)
UPD-596	Small molecule inhibitor	Naphthoquinone	Inhibits CDC25A	Kabakci et al. (2019)
UPD-1419	Small molecule inhibitor	Quinone	Inhibits CDC25A	Kabakci et al. (2019)
UPD-1416	Small molecule inhibitor	Quinone	Inhibits CDC25A	Kabakci et al. (2019)
UPD-795	Small molecule inhibitor	Naphthoquinone	Inhibits CDC25A	Kabakci et al. (2019)
Menadione	Small molecule inhibitor	Quinone	Inhibits CDC25A, B, C	Abdelwahab et al. (2022)
Cpd-5, [2-(2-mercaptoethanol)-3-methyl-1,4-naphthoquinone]	Small molecule inhibitor	Quinone	Inhibits CDC25A, B, C	Abdelwahab et al. (2022)
Cpd-42	Small molecule inhibitor	Vitamin K derivative	Inhibits CDC25A	Abdelwahab et al. (2022)
Cpd-5, derivative 6	Small molecule inhibitor	Vitamin K derivative	Inhibits CDC25A, B, C	Abdelwahab et al. (2022)
Cpd-5, isomer 7	Small molecule inhibitor	Vitamin K derivative	Inhibits CDC25A, B, C	Abdelwahab et al. (2022)
NS1’ Protein	natural product, an indirect inhibitor	Japanese Encephalitis Virus	Inhibits CDC25C	Li et al. (2021)
SV37	Small molecule inhibitor	coumarin-quinone derivative	Inhibits CDC25B, C	Abdelwahab et al. (2022)
Caulibugulones A-F	natural product, direct inhibitor	marine bryozoan *Caulibugula intermis*	Inhibits CDC25B	Abdelwahab et al. (2022)
Albendazole	Small molecule inhibitor	Benzimidazole	Inhibits CDC25A	Di Fusco et al. (2020)
Shikonen	Natural product, direct inhibition	Naphthoquinone*, Lithospermum erythrorhizon*	Inhibits CDC25C	Zhang et al. (2019)
APE	Natural product, Indirect inhibition	Annurca apple, polyphenol extract	Upregulate phosphor-CDC25C	Martino et al. (2019)

The discovery of dysidiolides in 1994 marked the beginning of the discovery of natural and synthetic compounds that modulate CDC25 family proteins. Many compounds have been described in recent reviews, and more patents and studies have reported new interacting molecules for CDC25 phosphatase inhibitors. Much effort has been focused on CDC25 phosphatase inhibitors in the past 5 years (Lavecchia et al., 2010). Dysidiolides are part of the small molecule inhibitor group, inhibiting CDC25 by binding to their catalytic site. These small molecule inhibitors are usually found using pharmacokinetic modelling studies. Knowledge of the crystalline and genetic structure has been evaluated and confirmed, opening up avenues for these small molecule inhibitors to be found using complex modelling and kinetic energy studies using a specific isoform of CDC25 and a repository of chemicals from the National Cancer Institute (Lazo et al., 2002). These inhibitors become pillars from which derivatives are constructed, using them as a backbone which has shown functional effectiveness for exploration against a CDC25 structural query, as in the dysidiolide case (Koch et al., 2004; Shimazawa et al., 2004).

Multiple cellular mechanisms can be invoked when dealing with CDC25 inhibition. The action may be direct by binding to CDC25 hotspots (Lavecchia et al., 2010), or indirectly through upstream inhibition, extant phosphorylation of CDC25 species (Lu et al., 2012) halting cell cycle progression, and inducing apoptosis (Martino et al., 2019). In terms of upstream effectors, regulating kinases such as JNK in cancers that have been observed to have perturbed activity in various cancer tissue, especially the invasive triple negative MDA-MB-231 breast cancer cells, are targets for many exploratory research groups for shutting down cell cycle progression (Sur and Agrawal, 2016; Martino et al., 2019). Another critical anti-oncogenic cellular pathway that should be considered within the discovery of cell cycle inhibitors are those that additionally trigger apoptosis. Polyphenols that are found in apple skins demonstrated, namely, APE was found to not only arrest cell cycle in G2 phase in CDC25C dependent manner but triggered ROS dependent intrinsic and extrinsic apoptosis in MDA-MB-231 (Martino et al., 2019). Once verified by *in-vitro/in-vivo* assays CDC25 inhibitor backbones that are not specific should be investigated, rather than discarded as there are programs using single cell transcriptomics in development that integrate patient-derived data to inform on drug combinations, such as ComboSC and the work of Berlow et al. (Berlow et al., 2019; Tang et al., 2023)

Modelling for active site binding mediated inhibition is difficult in CDC25 due to its unique structure. CDC25 has a shallow active site region and the reactivity of the catalytic cysteine residue compound this issue of active site binding. As an alternative, attention has turned to identifying hotspots in the enzyme that are critical for interactions at the phosphatase-substrate interface. Thirteen residues in CDC25B were identified, and mutations in R488 and Y497 reduced both *in vitro* and *in vivo* dephosphorylation of CdK2-pTpY/CycA by Cdc25B. A deep pocket adjacent to the hotspots on CDC25B harbours amino acids essential for substrate-phosphatase interactions, making compounds that selectively bind in this pocket potentially effective in disrupting CDC25B enzyme activity (Lavecchia et al., 2010). General protein phosphatase inhibitors can be found to potentially sensitize tumors to immunotherapy and chemotherapy through targeting of larger conserved domains (Stanford and Bottini, 2023). Active sites of enzymes are not the only potential targets that can be modelled for, as has been shown in an analogous case of binding to dimerization or ligand binding sites (Vartak et al., 2023). When taking these sites into account it seems that the possibilities to affect cellular mechanisms are vastly more plentiful and potentially more fruitful than the tunnel vision of active site binding. Briefly, this allosteric targeting can be used in downstream targets of CDC25, such as cyclin E/CDK2 or CDK1 at Y15, thus preventing phosphatase and subsequent proliferation (Pellerano et al., 2017), although this may lead to adverse effects compared to targeting of the functionally narrow CDC25 (Chu et al., 2021).

### 8.2 Small molecule inhibitors

One of the earliest small molecule inhibitors that arose from these early searches was NSC-95397 which was reported to be a dual-phosphatase inhibitor (Lazo et al., 2002). NSC-95397, a para-naphthoquinone, was found to bind to all of the isoforms of CDC25 with a low IC_50_ for CDC25 in colon cancer cells of 9.9–18.6 µM. However, within this study, it was found that CDC25A expression was not downregulated compared to controls but was actually phosphorylating downstream ERK1/2 (Dubey et al., 2018). In the context of acute myeloid leukemia, CDC25 inhibitor NSC-95397 was shown to exert anti-proliferative effects on cell suspensions. Most likely due to the aforementioned cyclin/CDK inhibition in a cytogenic-state-dependent manner (Brenner et al., 2017). Suggesting that some of the established CDC25 inhibitors still require deeper validation to confirm and understand the mechanisms at play.

Similarly, NSC-663284 was found to inhibit in a similar magnitude to the traditional gemcitabine treatment in mouse model experiments, with an inhibitory dose of 5 mg/kg. The efficacy of this CDC25 inhibitor for use in treatment was suggested to be influenced by several factors inherent to the molecule and the reaction with the metabolic reactions within the cells (Guo et al., 2007) and toxicity to the surrounding tissue. For instance, the quinone class of organic compounds, of which NSC-663284 is a part, has many members that effectively target and inhibit the enzyme activity of cdc25, binding with Tyr428, Arg482, Thr547 and Ser549 according to chemical binding simulations, (Guo et al., 2007; Ge et al., 2017), along with the additive effect of generating ROS that damage DNA, damages cellular superstructures and halts the progression of the cell cycle (Njus et al., 2023). Within the context of NSC-663284 it has been hypothesized that the inhibitor undergoes editing in order to form the electrostatic bonds to exert its inhibitory effects (Ge et al., 2017), if this modelling proves to be true with experimental data, the metabolite/edited product can be used in patient specific metabolic profiling of single cell colonies to evaluate the efficacy of NSC-663284 (Martino et al., 2019; Wekking et al., 2023).

CDC25 inhibitors could work indirectly on the deactivation of CDC25 by using endogenous regulation pathways and overexpressing/activating them leading to downregulation of CDC25. IRC-083864 is a strong CDC25 family protein inhibitor with low nanomolar activity and no inhibitory action on other phosphatases. Preventing mitosis and boosting CDK1 phosphorylation significantly suppressed tumour cell growth and changed cell cycle progression. IRC-083864 also caused apoptosis in tumour cells produced as spheroids and inhibited the development of human MIA PaCa-2 and LNCaP xenografts in animal models. While greater doses resulted in animal body weight loss, no harm was seen at lower doses (Brenner et al., 2014). The strong potency and anti-tumour activity of IRC-083864 promotes its further development as a viable treatment for drug-resistant malignancies. Another mechanism that can be used is ROS-mediated damage which would lead to the deactivation of CDC25 by phosphorylation via CHK1/2 (Kristjánsdóttir and Rudolph, 2004; Sur and Agrawal, 2016). NSC 119915, an irreversible inhibitor of this vein of mechanics, creates intracellular ROS in cells and stops them in the G0/G1 stage and G2/M stage of the cell cycle by inhibiting the two CDC25A and CDC25B. In previous studies, Genistein as an inhibitor has demonstrated a restriction of K562 leukemia, PC-3 prostate cancer, and MCF-7 breast cancer cell line progression considerably (Brenner et al., 2014).

Many novel small molecule inhibitors have been synthesized working off of *in silico* modelling of CDC25 inhibitors, considering structural, steric and isoformic activity and interactions. An issue can arise where there are too many small molecule inhibitors that are formulated from these methods and arise from too narrow a scope, but this is probably a good problem to have in relation to relying on possible ineffective and “brute-force” generalized therapies. The issue can be circumvented by using high-throughput methods but will experience a bottleneck at the animal testing stage, an invaluable stage that cannot be replaced (Tanoli et al., 2021). Suggesting that inhibitors to CDC25 activity may not need to specifically target the CDC25 protein but can affect the physiological effects associated with CDC25 involving pathways including mitotic spindle assembly (Cazales et al., 2007). For instance, the novel inhibitor WG-391D tested in ovarian cancer mouse models, presented advantageous effects to inhibiting tumor growth by down-regulating CDC25B (Xiao et al., 2019).

### 8.3 Natural product

The utilization of natural products and plant extracts has become more significant in the field of cancer research, primarily due to their potential as supplementary treatments. These substances present unique opportunities for the development of innovative therapeutic approaches. The extensive array of bioactive chemicals found within this particular subject matter offers promising prospects for precise and focused interventions. The potential for enhanced cancer management lies in the utilization of the synergistic effects between natural medicines and conventional therapy (Al-Eisawi et al., 2022a; Al-Eisawi et al., 2022b; Eldesouki et al., 2022; Bou Malhab et al., 2023; Tarawneh et al., 2023).

Another indirect and small molecule inhibitor of CDC25, from natural components, is Genistein. Genistein, a tyrosine kinase inhibitor, activated p38 in human mammary epithelial cells, involved in the downregulation of CDC25C levels and phosphorylation of CDC2 leading to an arrest of the cell cycle at the G2/M checkpoint (Frey and Singletary, 2003). Activation of p38, a mitogen-activated protein kinase, is essential for genistein-mediated growth inhibition, although it is not the only requirement. Additionally, genistein induces a G2 arrest by impairing the Tyr15 dephosphorylation of CDC2 via CDC25C, likely through a genistein-induced activation of CHK2 (Ouyang et al., 2009). Moreover, downregulation of the CDC25 level through p38 participation may be an important way to impair its actions and a meaningful act in G2/M checkpoint regulation. The investigation of the activities underlying the genistein inhibition or other agents on proliferation will need further clarification, considering the responses of other mitogen-activated protein kinase pathways, especially when considering different cytological sources (Frey and Singletary, 2003).

Even though there are beneficial result for single target CDC25 natural product inhibitors there is a greater need in our opinion for a multifaceted inhibitory attack on multiple isoforms of CDC25, that does not lose any specificity. In this regard natural products, such as Shikonin, could be of greater utility as observed in other phosphatase inhibitor combinations accompanying treatment granted that there is synergy and not agonistic effects (Marciniak et al., 2023). The research shows that the target effect of cell cycle checkpoint inhibitors may be expanded to inducing apoptosis. In the case of metabolite polyphenols APE retrieved from apple skins it was found to induce both apoptotic pathways, observed in the depletion of procaspase 3, 8 and 9, as well as arresting the cell cycle in a mimic to CDC25C action inhibition. However the main mode of action of the increase in inactivate phosphor-CDC25C was through ROS mediated cellular pathways indirectly acting on cell cycle progression rather than directly on CDC25 as is seen with most of the small molecule inhibitors (Martino et al., 2019). Offering additional anti-neoplastic effects that can be implemented in patient data driven combinatorial therapies, mainly through single cell metabolic profiling or a multiplexed omics platform which have been implemented with combinatorial therapies for ROS inducing agents (Huttunen et al., 2023) or immune checkpoint inhibitors (Massa et al., 2022). Given the results in other types of cellular pathway enducers and inhibitors there is hope that these results can also be translated to cell cycle checkpoint inhibitors such as CDC25 for sub-groups of patients identified as susceptible to the treatment.

### 8.4 Antisense oligonucleotides

In contrast, antisense oligonucleotides target the CDC25 mRNA and block its expression and synthesis much like the siRNA or miRNA mechanism of downregulation of translation. This is beyond the scope of this review, but the research shows that antisense oligonucleotides of CDC25 are effective in inhibiting cell cycle continuation through G1 to S. However, within the review search, it appears that researchers are studying affecting or upstream proteins to CDC25 which may suggest that post-transcriptional silencing of CDC25 directly is not a viable path for therapeutics due to the penetration of the silencing to the cell cycle. In theory, this could work but would require accurate and reliable delivery systems to specifically target neoplastic tissue. Allowing silencing through these means opens up combinatorial avenues of treatment with existing methods (Gharaibeh et al., 2021). One of the many advantages of using relatively novel strategies when it comes to tackling cancer is the hope that it may reduce multiple-drug *resistance* that can otherwise be observed with the more traditional and long-used chemotherapy cocktail of drugs. In *Xenopus laevis* studies of CDC25A antisense oligonucleotides, eggs injected with the oligonucleotide were found to prolong the presence of the repressed phosphorylated Cdk1 in a dose-dependent manner up to a threshold concentration (Yoshitome et al., 2019), suggestive of a potential therapeutic dose for inhibiting aberrant CDC25A activity. Further explored in hepatocellular carcinoma where the overexpression was related to poor prognosis, CDC25A antisense oligo demonstrated the ability to decrease invasiveness but more importantly reduced cell proliferation and progression (Xu et al., 2008). Although from the research articles found this field is not being explored as much recently relative to the aforementioned inhibitor types.

## 9 Challenges and future directions

### 9.1 Addressing challenges in developing specific and effective CDC25 inhibitors

However, the development of CDC25 inhibitors as a cancer treatment has been challenged by several limitations and obstacles. One of the challenges in the development of CDC25 inhibitors is the specificity of the inhibitors for different copies of CDC25 and their functions in maintaining the integrity of DNA on top of the cell cycle progression. CDC25A, CDC25B, and CDC25C have slightly different roles in the cell cycle; CD25A is involved in the G1/S transition activating the CDK2 promoting DNA replication entry in S-phase, CDC25B Therefore, it is important to develop inhibitors that are specific for each isoform of CDC25 to minimize off-target effects. A related challenge arises from the potential toxicity of CDC25 inhibitors due to the role CDC25 has in the regulation of the cell cycle, inhibiting its activity and leading to cell death. However, this can also affect normal cells and tissues, leading to side effects such as bone marrow suppression, gastrointestinal toxicity, and neurotoxicity. Therefore, it is important to develop CDC25 inhibitors that are selective for cancer cells and have minimal toxicity to normal cells.

Research has shown that the inherent structure and mechanism of action of CDC25 could also play a role in limiting the efficacy of CDC25 inhibitors. For one their broad substrate specificity poses a huge issue in terms of off-target effects. Dual specificity phosphatases (DSP) such as CDC25 can dephosphorylate a wide range of substrates, including kinases and phosphatases. This makes it challenging to design inhibitors that are specific to a particular DSP, without affecting other enzymes. The more broad-acting DSP would lead to the disruption of Mitogen-activated protein kinase phosphatase, Protein tyrosine phosphatase and Vaccinia H1-related phosphatase activities. Disruption of these DSPs can lead to irregularities in EGFR-initiated cell signalling cascades which could in fact lead to cancers (Wang et al., 2011). The structure of CDC25 as a DSP leads to difficulty in finding new potent inhibitors, arising from three obstacles presented by the active site on the Cdc25 phosphatase: the shallow active site region, the highly reactive cysteine in the active site, and the lack of homology with other protein phosphatases (Lavecchia et al., 2010).

To date there are not many CDC25 inhibitors that have shown significant results in immunosuppressed xenografted mouse models, and of the few I have mentioned the most relevant ones in terms of cancer therapeutics. The most notable to reach phase II of clinical trials was Debio 0931, the licensing name for IRC 083864 in 2009 but there is no news of if they have succeeded to phase III or require more confirmatory results. This reflects the lack of efficacy and displays the lack of translation of the *in-vitro* results to *in-vivo* and may be a significant drawback for using CDC25 inhibitors for cancer therapeutics. The promising technologies of omics and machine learning modules in biological chemistry seem to hold promise for identifying new molecules and derivatives that may be more successful than the current inventory of CDC25 inhibitors. Using some of the more chemically involved algorithms novel and synergetic CDC25 inhibitors have been found that demonstrate inhibition at the protein level (Lauria et al., 2021).

The repertoire of functional cell cycle checkpoint inhibitors is further expanded with the development of chemically inexpensive techniques, namely, click-chemistry, to build off putative backbones that demonstrate functional inhibitory activity. Constructive techniques have proven effective for radioligand imaging for immune checkpoint inhibitors, translation of this development for use in developing in cell cycle check-point inhibitors is not farfetched as reported in non-small cell lung cancer (Vartak et al., 2023). The power of which is expanded through integration of such formulation techniques with chemical library searching (Kabakci et al., 2019) prioritizing scaffolds, such as the quinone backbone, that show functional cell cycle inhibition *in-vitro* demonstrated in NSC663284 in colorectal cancer (Narwanti et al., 2023). The *in-vitro* testing is crucial as it can and has been used to validate *in silico* modelled compounds in other case types for anti-cancer effects (Berlow et al., 2019), which may slow down discovery but increases validity when combined with genes or gene products identified from omics techniques that are subsequently modelled for inhibitors (Chua et al., 2021; Wu Y. et al., 2024).

### 9.2 Exploring combination therapies involving CDC25 inhibitors

There are a growing number of studies that present personalized treatment with combination therapies improving outcomes in patients with refractory malignancies (Sicklick et al., 2019). Multimodal therapies have been observed to stratify risk, have stronger chemotherapy, and multimodality treatment strategies have significantly improved the mortality for children with cancer. Initial *in vitro* results showed additive and synergistic effects with other cell cycle inhibitors indirectly, or directly in the CDC25-specific inhibition paradigm that proved promising in their anti-proliferative action on various cancer cell lines (Larsson et al., 2009; Ock and Kim, 2021; Zhao et al., 2023). However, more improvement in survival rates and a decrease in long-term negative effects are required (Forrest et al., 2018). Many studies are coming to the same conclusion that combinatorial methods encompassing genomics, transcriptomics, and pathological images have improved prognostic models for various cancer types (Shao et al., 2023).

These methods have been involved with biomarkers not too dissimilar to the cell cycle marker CDC25 in cancers affecting large swathes of the population, including oesophageal squamous cell carcinoma (Liu et al., 2023), breast cancer (Greenwalt et al., 2020) and colorectal and bladder cancers (Ciardiello et al., 2022; Kong et al., 2022) and gastric cancers (Lee et al., 2019). Growing numbers of studies have pushed this field of study into clinal trials with promising results, as was seen with the gastric cancer umbrella study and in subsets of larger precision oncology clinical trials (O’Dwyer et al., 2023; Rodon et al., 2019) not only showing whether a particular clinical trial for a therapy is effective but by virtue of testing increasing knowledge of molecular significance of tumors; HER2 amplification for instance was found at a frequency of 2% in multiple tumors, not including breast cancers and gastric cancers (Jhaveri et al., 2019). However, analysis from other trials, in which specific biomarkers like CDKN2A, KRAS, and PIK3CA were identified, has shown that these methods may still provide limited results and require further development before entering regular patient processing procedures (Trédan et al., 2019). Clinical trials may seem to lag on the potential of personalizing treatments with regard to aberrant cell cycle control checkpoints.

The synergy between click chemistry methodologies and the conjugation of small molecule inhibitors to monoclonal antibodies represents an innovative Frontier in personalized cancer therapy within the realm of precision medicine. Click chemistry’s precision in molecular design and modification allows for the customization of small molecule inhibitors, enabling their conjugation to monoclonal antibodies tailored to individual patients’ molecular profiles. Additionally, the involvement of mathematical modelling as well as machine learning modalities can expand potential useful conjugates (Pang et al., 2023). This personalized and *in silico* refined approach facilitates the development of targeted therapeutic agents designed to recognize and bind with high specificity to unique antigens or surface markers present on an individual’s cancer cells (Vartak et al., 2023; Greenlee et al., 2024). By leveraging the specificity of monoclonal antibodies for these patient-specific molecular signatures, click chemistry empowers the creation of highly personalized multifunctional agents capable of precisely delivering the small molecule inhibitors to the patient’s specific cancerous lesions. This personalized targeting strategy holds immense promise in tailored cancer therapy within the framework of precision medicine, offering a bespoke and targeted therapeutic avenue by harnessing the amalgamation of small molecule inhibitors, monoclonal antibodies, and individualized molecular characteristics for enhanced treatment outcomes.

Personalized medicine data along with advanced chemical-focused exploratory algorithms (Rifaioglu et al., 2019) can direct combination therapies based on novel assay methods working through databases in a high-throughput manner (Lauria et al., 2021). Artificial intelligence along with traditional methods of immunotherapy and chemotherapy have preliminarily shown beneficial prognosis for cancer patients based on results from *in-vitro* assays and limited clinical trials (Kuenzi et al., 2020). Identifying and typing cancerous tissue from benign tissue have been investigated more than the clinical trials, AI models have been developed to predict RNA-Seq profiles and MSI in various cancer types, incorporating multimodal data and transfer learning for improved predictions in various cancers including breast, gastrointestinal and colorectal cancer types (Shao et al., 2023). In endometrial cancer a variety of combinatorial treatments were tested in clinical trials and applied based on molecular markers found within patients’ malignant tissue, increasing late-stage patients from 40% to almost 80% survival after more precise (Arend et al., 2018). Combining molecular omics and radiomics (Avanzo et al., 2020) can potentially increase the effectiveness of targeted radiotherapy by reducing and eliminating patients that would prove unsuccessful or remittent to such an approach.

## 10 Conclusion

Ultimately the evidence reveals that CDC25 inhibitors have tremendous potential as a therapeutic target for cancer therapy, but we would not recommend it as complete but may be part of a greater whole to additionally target in combinatorial therapy. These inhibitors, by modulating CDC25 activity, can impede CDK dephosphorylation and subsequent cell cycle progression, resulting in cell cycle arrest, apoptosis, and decreased tumour development. Preclinical investigations using numerous CDC25 inhibitors, including small molecules, peptides, and natural substances, have yielded encouraging findings, exhibiting significant antitumor action in several cancer types. Clinical trials examining the safety and effectiveness of these inhibitors as monotherapy or in combination with other anticancer treatments, however, are still in the early stages and should validate the results found in the many *in-vitro* studies. Furthermore, the possible toxicity and off-target consequences of these inhibitors should be explored further. The combinatorial treatment high throughput information driven paradigm alongside comprehensive CDC25 inhibitors are some of the most promising directions in halting cell cycle progression in cancer and should garner more focus with regards to clinical trials, even though they may be more difficult to design. While the development of CDC25 inhibitors as a cancer therapy method is still in its early stages and has faced some obstacles, data shows that this technique has tremendous potential and needs further exploration.

## References

[B1] Abdelwahab A. B. El-Sawy E. R. Hanna A. G. Bagrel D. Kirsch G. (2022). A comprehensive overview of the developments of Cdc25 phosphatase inhibitors. Molecules 27 (8). 10.3390/molecules27082389 PMC9031484 35458583

[B2] Abushawish K. Y. I. Soliman S. S. M. Giddey A. D. Al-Hroub H. M. Mousa M. Alzoubi K. H. (2022). Multi-omics analysis revealed a significant alteration of critical metabolic pathways due to sorafenib-resistance in Hep3B cell lines. Int. J. Mol. Sci. 23 (19), 11975. 10.3390/ijms231911975 36233276 PMC9569810

[B3] Agius E. Bel-Vialar S. Bonnet F. Pituello F. (2015). Cell cycle and cell fate in the developing nervous system: the role of CDC25B phosphatase. Cell Tissue Res. 359 (1), 201–213. 10.1007/s00441-014-1998-2 25260908

[B4] Ahmed M. Semreen A. M. El-Huneidi W. Bustanji Y. Abu-Gharbieh E. Alqudah M. A. Y. (2023). Preclinical and clinical applications of metabolomics and proteomics in glioblastoma research [review]. Int. J. Mol. Sci. 24 (1), 348. 10.3390/ijms24010348 PMC9820403 36613792

[B5] Al-Eisawi Z. Abderrahman S. M. Abdelrahim Y. M. S. Al-Abbassi R. Bustanji Y. K. (2022a). Anastatica hierochuntica extracts: promising, safe and selective anti-cancer agents. Nat. Prod. J. 12 (1), e160921185898. 10.2174/2210315510999200914153725

[B6] Al-Eisawi Z. Abderrahman S. M. Al-Khalaf I. F. Al-Abbassi R. Bustanji Y. K. (2022b). *Taraxacum officinale* extracts exhibit safe and selective anticancer activity [article]. Nat. Prod. J. 12 (1), e160921187741. 10.2174/2210315510999201109202255

[B7] Alfarouk K. O. Stock C. M. Taylor S. Walsh M. Muddathir A. K. Verduzco D. (2015). Resistance to cancer chemotherapy: failure in drug response from ADME to P-gp. Cancer Cell Int. 15, 71. 10.1186/s12935-015-0221-1 26180516 PMC4502609

[B8] Arend R. C. Jones B. A. Martinez A. Goodfellow P. (2018). Endometrial cancer: molecular markers and management of advanced stage disease. Gynecol. Oncol. 150 (3), 569–580. 10.1016/j.ygyno.2018.05.015 29843906

[B9] Avanzo M. Stancanello J. Pirrone G. Sartor G. (2020). Radiomics and deep learning in lung cancer. Strahlenther Onkol. 196 (10), 879–887. 10.1007/s00066-020-01625-9 32367456

[B10] Axelrod M. L. Johnson D. B. Balko J. M. (2018). Emerging biomarkers for cancer immunotherapy in melanoma. Semin. Cancer Biol. 52 (Pt 2), 207–215. 10.1016/j.semcancer.2017.09.004 28917578 PMC5851807

[B11] Baldin V. Cans C. Superti-Furga G. Ducommun B. (1997). Alternative splicing of the human CDC25B tyrosine phosphatase. Possible implications for growth control? Oncogene 14 (20), 2485–2495. 10.1038/sj.onc.1201063 9188863

[B12] Banchereau R. Leng N. Zill O. Sokol E. Liu G. Pavlick D. (2021). Molecular determinants of response to PD-L1 blockade across tumor types. Nat. Commun. 12 (1), 3969. 10.1038/s41467-021-24112-w 34172722 PMC8233428

[B13] Berlow N. E. Rikhi R. Geltzeiler M. Abraham J. Svalina M. N. Davis L. E. (2019). Probabilistic modeling of personalized drug combinations from integrated chemical screen and molecular data in sarcoma. BMC Cancer 19 (1), 593. 10.1186/s12885-019-5681-6 31208434 PMC6580486

[B14] Bhinder B. Gilvary C. Madhukar N. S. Elemento O. (2021). Artificial intelligence in cancer research and precision medicine. Cancer Discov. 11 (4), 900–915. 10.1158/2159-8290.Cd-21-0090 33811123 PMC8034385

[B15] Bou Malhab L. J. Bajbouj K. Shehab N. G. Elayoty S. M. Sinoj J. Adra S. (2023). Potential anticancer properties of calotropis procera: an investigation on breast and colon cancer cells. Heliyon 9 (6), e16706. 10.1016/j.heliyon.2023.e16706 37332907 PMC10272338

[B16] Boutros R. Dozier C. Ducommun B. (2006). The when and wheres of CDC25 phosphatases. Curr. Opin. Cell Biol. 18 (2), 185–191. 10.1016/j.ceb.2006.02.003 16488126

[B17] Brenner A. K. Reikvam H. Lavecchia A. Bruserud Ø. (2014). Therapeutic targeting the cell division cycle 25 (CDC25) phosphatases in human acute myeloid leukemia--the possibility to target several kinases through inhibition of the various CDC25 isoforms. Molecules 19 (11), 18414–18447. 10.3390/molecules191118414 25397735 PMC6270710

[B18] Brenner A. K. Reikvam H. Rye K. P. Hagen K. M. Lavecchia A. Bruserud Ø. (2017). CDC25 inhibition in acute myeloid leukemia-A study of patient heterogeneity and the effects of different inhibitors. Molecules 22 (3). 10.3390/molecules22030446 PMC6155411 28287460

[B19] Bretones G. Delgado M. D. León J. (2015). Myc and cell cycle control. Biochim. Biophys. Acta 1849 (5), 506–516. 10.1016/j.bbagrm.2014.03.013 24704206

[B20] Brezak M. C. Valette A. Quaranta M. Contour-Galcera M. O. Jullien D. Lavergne O. (2009). IRC-083864, a novel bis quinone inhibitor of CDC25 phosphatases active against human cancer cells. Int. J. Cancer 124 (6), 1449–1456. 10.1002/ijc.24080 19065668

[B21] Broggini M. Buraggi G. Brenna A. Riva L. Codegoni A. M. Torri V. (2000). Cell cycle-related phosphatases CDC25A and B expression correlates with survival in ovarian cancer patients. Anticancer Res. 20 (6c), 4835–4840. 11205229

[B22] Cangi M. G. Cukor B. Soung P. Signoretti S. Moreira G. Jr. Ranashinge M. (2000). Role of the Cdc25A phosphatase in human breast cancer. J. Clin. Invest. 106 (6), 753–761. 10.1172/jci9174 10995786 PMC381390

[B23] Carapito Â. Roque A. C. A. Carvalho F. Pinto J. Guedes de Pinho P. (2024). Exploiting volatile fingerprints for bladder cancer diagnosis: a scoping review of metabolomics and sensor-based approaches. Talanta, 268, 125296. 10.1016/j.talanta.2023.125296 37839328

[B24] Cazales M. Boutros R. Brezak M. C. Chaumeron S. Prevost G. Ducommun B. (2007). Pharmacologic inhibition of CDC25 phosphatases impairs interphase microtubule dynamics and mitotic spindle assembly. Mol. Cancer Ther. 6 (1), 318–325. 10.1158/1535-7163.Mct-06-0299 17237290

[B25] Chen Z. H. Lin L. Wu C. F. Li C. F. Xu R. H. Sun Y. (2021). Artificial intelligence for assisting cancer diagnosis and treatment in the era of precision medicine. Cancer Commun. (Lond) 41 (11), 1100–1115. 10.1002/cac2.12215 34613667 PMC8626610

[B26] Chowdhary S. Raza A. PreetiKaur S. Anand A. Sharma A. K. Kumar V. (2024). Isatin-indoloquinoxaline click adducts with a potential to overcome platinum-based drug-resistance in ovarian cancer. Bioorg Chem. 142, 106953. 10.1016/j.bioorg.2023.106953 37925887

[B27] Chu C. Geng Y. Zhou Y. Sicinski P. (2021). Cyclin E in normal physiology and disease states. Trends Cell Biol. 31 (9), 732–746. 10.1016/j.tcb.2021.05.001 34052101 PMC8364501

[B28] Chua I. S. Gaziel-Yablowitz M. Korach Z. T. Kehl K. L. Levitan N. A. Arriaga Y. E. (2021). Artificial intelligence in oncology: path to implementation. Cancer Med. 10 (12), 4138–4149. 10.1002/cam4.3935 33960708 PMC8209596

[B29] Ciardiello F. Ciardiello D. Martini G. Napolitano S. Tabernero J. Cervantes A. (2022). Clinical management of metastatic colorectal cancer in the era of precision medicine. CA Cancer J. Clin. 72 (4), 372–401. 10.3322/caac.21728 35472088

[B30] Cristescu R. Mogg R. Ayers M. Albright A. Murphy E. Yearley J. (2018). Pan-tumor genomic biomarkers for PD-1 checkpoint blockade-based immunotherapy. Science 362 (6411). 10.1126/science.aar3593 PMC6718162 30309915

[B31] Cyriac R. Lee K. (2024). Glutaminase inhibition as potential cancer therapeutics: current status and future applications. J. Enzyme Inhib. Med. Chem. 39 (1), 2290911. 10.1080/14756366.2023.2290911 38078371 PMC11721875

[B32] Dahabiyeh L. A. Hourani W. Darwish W. Hudaib F. Abu-Irmaileh B. Deb P. K. (2022). Molecular and metabolic alterations of 2,3-dihydroquinazolin-4(1H)-one derivatives in prostate cancer cell lines. Sci. Rep. 12 (1), 21599. 10.1038/s41598-022-26148-4 36517571 PMC9751122

[B33] Dahabiyeh L. A. Hudaib F. Hourani W. Darwish W. Abu-Irmaileh B. Deb P. K. (2023). Mass spectrometry-based metabolomics approach and *in vitro* assays revealed promising role of 2,3-dihydroquinazolin-4(1H)-one derivatives against colorectal cancer cell lines. Eur. J. Pharm. Sci. 182, 106378. 10.1016/j.ejps.2023.106378 36638899

[B34] Dai X. Shi X. Luo M. Li P. Gao Y. (2023). Integrative analysis of transcriptomic and metabolomic profiles reveals enhanced arginine metabolism in androgen-independent prostate cancer cells. BMC Cancer 23 (1), 1241. 10.1186/s12885-023-11707-3 38104097 PMC10724921

[B35] Di Fusco D. Stolfi C. Di Grazia A. Dinallo V. Laudisi F. Marafini I. (2020). Albendazole negatively regulates keratinocyte proliferation. Clin. Sci. (Lond) 134 (7), 907–920. 10.1042/cs20191215 32236445

[B36] Ditano J. P. Sakurikar N. Eastman A. (2021). Activation of CDC25A phosphatase is limited by CDK2/cyclin A-mediated feedback inhibition. Cell Cycle 20 (13), 1308–1319. 10.1080/15384101.2021.1938813 34156324 PMC8331022

[B37] Donzelli M. Squatrito M. Ganoth D. Hershko A. Pagano M. Draetta G. F. (2002). Dual mode of degradation of Cdc25 A phosphatase. Embo J. 21 (18), 4875–4884. 10.1093/emboj/cdf491 12234927 PMC126287

[B38] Dubey N. K. Peng B. Y. Lin C. M. Wang P. D. Wang J. R. Chan C. H. (2018). NSC 95397 suppresses proliferation and induces apoptosis in colon cancer cells through MKP-1 and the ERK1/2 pathway. Int. J. Mol. Sci. 19 (6). 10.3390/ijms19061625 PMC6032145 29857489

[B39] Eldesouki S. Qadri R. Abu Helwa R. Barqawi H. Bustanji Y. Abu-Gharbieh E. (2022). Recent updates on the functional impact of kahweol and cafestol on cancer [review]. Molecules 27 (21), 7332. 10.3390/molecules27217332 36364160 PMC9654648

[B40] Forrest S. J. Geoerger B. Janeway K. A. (2018). Precision medicine in pediatric oncology. Curr. Opin. Pediatr. 30 (1), 17–24. 10.1097/mop.0000000000000570 29189430 PMC5770114

[B41] Frazer C. Young P. G. (2012). “Phosphorylation mediated regulation of Cdc25 activity, localization and stability,” in Protein phosphorylation in human health. Editor Cai H. (IntechOpen). Ch. 14). 10.5772/48315

[B42] Frey R. S. Singletary K. W. (2003). Genistein activates p38 mitogen-activated protein kinase, inactivates ERK1/ERK2 and decreases Cdc25C expression in immortalized human mammary epithelial cells. J. Nutr. 133 (1), 226–231. 10.1093/jn/133.1.226 12514295

[B43] Gabrielli B. G. De Souza C. P. Tonks I. D. Clark J. M. Hayward N. K. Ellem K. A. (1996). Cytoplasmic accumulation of cdc25B phosphatase in mitosis triggers centrosomal microtubule nucleation in HeLa cells. J. Cell Sci. 109 (Pt 5), 1081–1093. 10.1242/jcs.109.5.1081 8743955

[B44] Galaktionov K. Chen X. Beach D. (1996). Cdc25 cell-cycle phosphatase as a target of c-myc. Nature 382 (6591), 511–517. 10.1038/382511a0 8700224

[B45] Garrido-Castro A. C. Lin N. U. Polyak K. (2019). Insights into molecular classifications of triple-negative breast cancer: improving patient selection for treatment. Cancer Discov. 9 (2), 176–198. 10.1158/2159-8290.Cd-18-1177 30679171 PMC6387871

[B46] Ge Y. van der Kamp M. Malaisree M. Liu D. Liu Y. Mulholland A. J. (2017). Identification of the quinolinedione inhibitor binding site in Cdc25 phosphatase B through docking and molecular dynamics simulations. J. Comput. Aided Mol. Des. 31 (11), 995–1007. 10.1007/s10822-017-0073-y 28994029

[B47] Gharaibeh L. Alshaer W. Wehaibi S. Al Buqain R. Alqudah D. A. Al-Kadash A. (2021). Fabrication of aptamer-guided siRNA loaded lipopolyplexes for gene silencing of notch 1 in MDA-mb-231 triple negative breast cancer cell line. J. Drug Deliv. Sci. Technol. 65, 102733. 10.1016/j.jddst.2021.102733

[B48] Greenlee J. D. Zhang Z. Subramanian T. Liu K. King M. R. (2024). TRAIL-conjugated liposomes that bind natural killer cells to induce colorectal cancer cell apoptosis. J. Biomed. Mater Res. A 112 (1), 110–120. 10.1002/jbm.a.37621 37772330 PMC10794038

[B49] Greenwalt I. Zaza N. Das S. Li B. D. (2020). Precision medicine and targeted therapies in breast cancer. Surg. Oncol. Clin. N. Am. 29 (1), 51–62. 10.1016/j.soc.2019.08.004 31757313

[B50] Guo J. Parise R. A. Joseph E. Lan J. Pan S. S. Joo B. (2007). Pharmacology and antitumor activity of a quinolinedione Cdc25 phosphatase inhibitor DA3003-1 (NSC 663284). Anticancer Res. 27 (5a), 3067–3073. 17970046

[B51] Hagyousif Y. A. Sharaf B. M. Zenati R. A. El-Huneidi W. Bustanji Y. Abu-Gharbieh E. (2023). Skin cancer metabolic profile assessed by different analytical platforms [review]. Int. J. Mol. Sci. 24 (2), 1604. 10.3390/ijms24021604 36675128 PMC9866771

[B52] Hainsworth J. D. Meric-Bernstam F. Swanton C. Hurwitz H. Spigel D. R. Sweeney C. (2018). Targeted therapy for advanced solid tumors on the basis of molecular profiles: results from MyPathway, an open-label, phase IIa multiple basket study. J. Clin. Oncol. 36 (6), 536–542. 10.1200/jco.2017.75.3780 29320312

[B53] He B. Dong D. She Y. Zhou C. Fang M. Zhu Y. (2020). Predicting response to immunotherapy in advanced non-small-cell lung cancer using tumor mutational burden radiomic biomarker. J. Immunother. Cancer 8 (2). 10.1136/jitc-2020-000550 PMC7342823 32636239

[B54] He R. Zeng L. F. He Y. Zhang S. Zhang Z. Y. (2013). Small molecule tools for functional interrogation of protein tyrosine phosphatases. Febs J. 280 (2), 731–750. 10.1111/j.1742-4658.2012.08718.x 22816879 PMC3495087

[B55] He Y. Wang X. (2023). Identifying biomarkers associated with immunotherapy response in melanoma by multi-omics analysis. Comput. Biol. Med. 167, 107591. 10.1016/j.compbiomed.2023.107591 37875043

[B56] Hernández S. Hernández L. Beà S. Cazorla M. Fernández P. L. Nadal A. (1998). cdc25 cell cycle-activating phosphatases and c-myc expression in human non-Hodgkin's lymphomas. Cancer Res. 58 (8), 1762–1767. 9563496

[B57] Hoffmann I. Karsenti E. (1994). The role of cdc25 in checkpoints and feedback controls in the eukaryotic cell cycle. J. Cell Sci. Suppl. 18, 75–79. 10.1242/jcs.1994.supplement_18.11 7883797

[B58] Huttunen J. Tampio J. Järvinen J. Montaser A. B. Markowicz-Piasecka M. Huttunen K. M. (2023). Amino acid derivative of probenecid potentiates apoptosis-inducing effects of vinblastine by increasing oxidative stress in a cancer cell-specific manner. Chemico-Biological Interact., 110833. 10.1016/j.cbi.2023.110833 38101600

[B59] Issa N. T. Stathias V. Schürer S. Dakshanamurthy S. (2021). Machine and deep learning approaches for cancer drug repurposing. Semin. Cancer Biol. 68, 132–142. 10.1016/j.semcancer.2019.12.011 31904426 PMC7723306

[B60] Jhaveri K. L. Wang X. V. Makker V. Luoh S. W. Mitchell E. P. Zwiebel J. A. (2019). Ado-trastuzumab emtansine (T-DM1) in patients with HER2-amplified tumors excluding breast and gastric/gastroesophageal junction (GEJ) adenocarcinomas: results from the NCI-MATCH trial (EAY131) subprotocol Q. Ann. Oncol. 30 (11), 1821–1830. 10.1093/annonc/mdz291 31504139 PMC6927318

[B61] Johannet P. Coudray N. Donnelly D. M. Jour G. Illa-Bochaca I. Xia Y. (2021). Using machine learning algorithms to predict immunotherapy response in patients with advanced melanoma. Clin. Cancer Res. 27 (1), 131–140. 10.1158/1078-0432.Ccr-20-2415 33208341 PMC7785656

[B62] Kabakci Z. Käppeli S. Cantù C. Jensen L. D. König C. Toggweiler J. (2019). Pharmacophore-guided discovery of CDC25 inhibitors causing cell cycle arrest and tumor regression. Sci. Rep. 9 (1), 1335. 10.1038/s41598-019-38579-7 30718768 PMC6362118

[B63] Koch M. A. Wittenberg L. O. Basu S. Jeyaraj D. A. Gourzoulidou E. Reinecke K. (2004). Compound library development guided by protein structure similarity clustering and natural product structure. Proc. Natl. Acad. Sci. U. S. A. 101 (48), 16721–16726. 10.1073/pnas.0404719101 15548605 PMC534721

[B64] Kong J. Ha D. Lee J. Kim I. Park M. Im S. H. (2022). Network-based machine learning approach to predict immunotherapy response in cancer patients. Nat. Commun. 13 (1), 3703. 10.1038/s41467-022-31535-6 35764641 PMC9240063

[B65] Kristjánsdóttir K. Rudolph J. (2004). Cdc25 phosphatases and cancer. Chem. Biol. 11 (8), 1043–1051. 10.1016/j.chembiol.2004.07.007 15324805

[B66] Kuenzi B. M. Park J. Fong S. H. Sanchez K. S. Lee J. Kreisberg J. F. (2020). Predicting drug response and synergy using a deep learning model of human cancer cells. Cancer Cell 38 (5), 672–684. 10.1016/j.ccell.2020.09.014 33096023 PMC7737474

[B67] Larsson D. E. Hassan S. Larsson R. Oberg K. Granberg D. (2009). Combination analyses of anti-cancer drugs on human neuroendocrine tumor cell lines. Cancer Chemother. Pharmacol. 65 (1), 5–12. 10.1007/s00280-009-0997-6 19381631

[B68] Lauria A. Martorana A. La Monica G. Mannino S. Mannino G. Peri D. (2021). *In silico* identification of small molecules as new Cdc25 inhibitors through the correlation between chemosensitivity and protein expression pattern. Int. J. Mol. Sci. 22 (7). 10.3390/ijms22073714 PMC8038176 33918281

[B69] Lavecchia A. Di Giovanni C. Novellino E. (2009). CDC25A and B dual-specificity phosphatase inhibitors: potential agents for cancer therapy. Curr. Med. Chem. 16 (15), 1831–1849. 10.2174/092986709788186084 19442149

[B70] Lavecchia A. Di Giovanni C. Novellino E. (2010). Inhibitors of Cdc25 phosphatases as anticancer agents: a patent review. Expert Opin. Ther. Pat. 20 (3), 405–425. 10.1517/13543771003623232 20166845

[B71] Lazo J. S. Nemoto K. Pestell K. E. Cooley K. Southwick E. C. Mitchell D. A. (2002). Identification of a potent and selective pharmacophore for Cdc25 dual specificity phosphatase inhibitors. Mol. Pharmacol. 61 (4), 720–728. 10.1124/mol.61.4.720 11901209

[B72] Lee J. Kim S. T. Kim K. Lee H. Kozarewa I. Mortimer P. G. S. (2019). Tumor genomic profiling guides patients with metastatic gastric cancer to targeted treatment: the VIKTORY umbrella trial. Cancer Discov. 9 (10), 1388–1405. 10.1158/2159-8290.Cd-19-0442 31315834

[B73] Li J. Qiao H. Wu F. Sun S. Feng C. Li C. (2022). A novel hypoxia- and lactate metabolism-related signature to predict prognosis and immunotherapy responses for breast cancer by integrating machine learning and bioinformatic analyses. Front. Immunol. 13, 998140. 10.3389/fimmu.2022.998140 36275774 PMC9585224

[B74] Li Q. Zhou D. Jia F. Zhang L. Ashraf U. Li Y. (2021). Japanese encephalitis virus NS1' protein interacts with host CDK1 protein to regulate antiviral response. Microbiol. Spectr. 9 (3), e0166121. 10.1128/Spectrum.01661-21 34756071 PMC8579942

[B75] Lin L. Li Z. Yan L. Liu Y. Yang H. Li H. (2021). Global, regional, and national cancer incidence and death for 29 cancer groups in 2019 and trends analysis of the global cancer burden, 1990-2019. J. Hematol. Oncol. 14 (1), 197. 10.1186/s13045-021-01213-z 34809683 PMC8607714

[B76] Liu J. C. Granieri L. Shrestha M. Wang D. Y. Vorobieva I. Rubie E. A. (2018). Identification of CDC25 as a common therapeutic target for triple-negative breast cancer. Cell Rep. 23 (1), 112–126. 10.1016/j.celrep.2018.03.039 29617654 PMC9357459

[B77] Liu K. Zheng M. Lu R. Du J. Zhao Q. Li Z. (2020). The role of CDC25C in cell cycle regulation and clinical cancer therapy: a systematic review. Cancer Cell Int. 20 (1), 213. 10.1186/s12935-020-01304-w 32518522 PMC7268735

[B78] Liu Z. Zhao Y. Kong P. Liu Y. Huang J. Xu E. (2023). Integrated multi-omics profiling yields a clinically relevant molecular classification for esophageal squamous cell carcinoma. Cancer Cell 41 (1), 181–195. 10.1016/j.ccell.2022.12.004 36584672

[B79] Löffler H. Syljuåsen R. G. Bartkova J. Worm J. Lukas J. Bartek J. (2003). Distinct modes of deregulation of the proto-oncogenic Cdc25A phosphatase in human breast cancer cell lines. Oncogene 22 (50), 8063–8071. 10.1038/sj.onc.1206976 14603247

[B80] Lu L. X. Domingo-Sananes M. R. Huzarska M. Novak B. Gould K. L. (2012). Multisite phosphoregulation of Cdc25 activity refines the mitotic entrance and exit switches. Proc. Natl. Acad. Sci. U. S. A. 109 (25), 9899–9904. 10.1073/pnas.1201366109 22665807 PMC3382524

[B81] Lund G. Dudkin S. Borkin D. Ni W. Grembecka J. Cierpicki T. (2015). Inhibition of CDC25B phosphatase through disruption of protein-protein interaction. ACS Chem. Biol. 10 (2), 390–394. 10.1021/cb500883h 25423142 PMC4340349

[B82] Malla M. Loree J. M. Kasi P. M. Parikh A. R. (2022). Using circulating tumor DNA in colorectal cancer: current and evolving practices. J. Clin. Oncol. 40 (24), 2846–2857. 10.1200/jco.21.02615 35839443 PMC9390824

[B83] Marciniak B. Kciuk M. Mujwar S. Sundaraj R. Bukowski K. Gruszka R. (2023). Vitro and *in silico* investigation of BCI anticancer properties and its potential for chemotherapy-combined treatments. Cancers (Basel) 15 (18). 10.3390/cancers15184442 PMC10526149 37760412

[B84] Martino E. Vuoso D. C. D'Angelo S. Mele L. D'Onofrio N. Porcelli M. (2019). Annurca apple polyphenol extract selectively kills MDA-MB-231 cells through ROS generation, sustained JNK activation and cell growth and survival inhibition. Sci. Rep. 9 (1), 13045. 10.1038/s41598-019-49631-x 31506575 PMC6736874

[B85] Massa D. Tosi A. Rosato A. Guarneri V. Dieci M. V. (2022). Multiplexed *in situ* spatial protein profiling in the pursuit of precision immuno-oncology for patients with breast cancer. Cancers (Basel) 14 (19). 10.3390/cancers14194885 PMC9562913 36230808

[B86] Mehlich D. Marusiak A. A. (2022). Kinase inhibitors for precision therapy of triple-negative breast cancer: progress, challenges, and new perspectives on targeting this heterogeneous disease. Cancer Lett. 547, 215775. 10.1016/j.canlet.2022.215775 35667515

[B87] Meijer J. J. Leonetti A. Airò G. Tiseo M. Rolfo C. Giovannetti E. (2022). Small cell lung cancer: novel treatments beyond immunotherapy. Semin. Cancer Biol. 86 (Pt 2), 376–385. 10.1016/j.semcancer.2022.05.004 35568295

[B88] Miyata H. Doki Y. Shiozaki H. Inoue M. Yano M. Fujiwara Y. (2000). CDC25B and p53 are independently implicated in radiation sensitivity for human esophageal cancers. Clin. Cancer Res. 6 (12), 4859–4865. 11156245

[B89] Monsivais D. Parks S. E. Chandrashekar D. S. Varambally S. Creighton C. J. (2023). Using cancer proteomics data to identify gene candidates for therapeutic targeting. Oncotarget 14, 399–412. 10.18632/oncotarget.28420 37141409 PMC11623401

[B90] Motawi T. M. K. Bustanji Y. El-Maraghy S. Taha M. O. Al-Ghussein M. A. S. (2014). Evaluation of naproxen and cromolyn activities against cancer cells viability, proliferation, apoptosis, p53 and gene expression of survivin and caspase-3. J. Enzyme Inhibition Med. Chem. 29 (2), 153–161. 10.3109/14756366.2012.762645 23368763

[B91] Nagpal K. Foote D. Tan F. Liu Y. Chen P. C. Steiner D. F. (2020). Development and validation of a deep learning algorithm for Gleason grading of prostate cancer from biopsy specimens. JAMA Oncol. 6 (9), 1372–1380. 10.1001/jamaoncol.2020.2485 32701148 PMC7378872

[B92] Naithani N. Sinha S. Misra P. Vasudevan B. Sahu R. (2021). Precision medicine: concept and tools. Med. J. Armed Forces India 77 (3), 249–257. 10.1016/j.mjafi.2021.06.021 34305276 PMC8282508

[B93] Nakano T. Chen C. L. Chen I. H. Tseng H. P. Chiang K. C. Lai C. Y. (2023). Overexpression of miR-4669 enhances tumor aggressiveness and generates an immunosuppressive tumor microenvironment in hepatocellular carcinoma: its clinical value as a predictive biomarker. Int. J. Mol. Sci. 24 (9). 10.3390/ijms24097908 PMC10177802 37175615

[B94] Narwanti I. Yu Z. Y. Sethy B. Lai M. J. Lee H. Y. Olena P. (2023). 6-Regioisomeric 5,8-quinolinediones as potent CDC25 inhibitors against colorectal cancers. Eur. J. Med. Chem. 258, 115505. 10.1016/j.ejmech.2023.115505 37302341

[B95] Nayarisseri A. Khandelwal R. Tanwar P. Madhavi M. Sharma D. Thakur G. (2021). Artificial intelligence, big data and machine learning approaches in precision medicine & drug discovery. Curr. Drug Targets 22 (6), 631–655. 10.2174/1389450122999210104205732 33397265

[B96] Nemoto K. Vogt A. Oguri T. Lazo J. S. (2004). Activation of the Raf-1/MEK/Erk kinase pathway by a novel Cdc25 inhibitor in human prostate cancer cells. Prostate 58 (1), 95–102. 10.1002/pros.10292 14673957

[B97] Njus D. Asmaro K. Li G. Palomino E. (2023). Redox cycling of quinones reduced by ascorbic acid. Chem. Biol. Interact. 373, 110397. 10.1016/j.cbi.2023.110397 36764370

[B98] Ock C. W. Kim G. D. (2021). Harmine hydrochloride mediates the induction of G2/M cell cycle arrest in breast cancer cells by regulating the MAPKs and AKT/FOXO3a signaling pathways. Molecules 26 (21). 10.3390/molecules26216714 PMC8588485 34771123

[B99] O'Dwyer P. J. Gray R. J. Flaherty K. T. Chen A. P. Li S. Wang V. (2023). The NCI-MATCH trial: lessons for precision oncology. Nat. Med. 29 (6), 1349–1357. 10.1038/s41591-023-02379-4 37322121 PMC10612141

[B100] Olivier M. Asmis R. Hawkins G. A. Howard T. D. Cox L. A. (2019). The need for multi-omics biomarker signatures in precision medicine. Int. J. Mol. Sci. 20 (19). 10.3390/ijms20194781 PMC6801754 31561483

[B101] Ouyang G. Yao L. Ruan K. Song G. Mao Y. Bao S. (2009). Genistein induces G2/M cell cycle arrest and apoptosis of human ovarian cancer cells via activation of DNA damage checkpoint pathways. Cell Biol. Int. 33 (12), 1237–1244. 10.1016/j.cellbi.2009.08.011 19732843

[B102] Pang J. Zhuang B. Zhang L. M. (2023). A co-carrier for plasmid DNA and curcumin delivery to treat pancreatic cancer via dendritic poly(l-lysine) modified amylose. Int. J. Biol. Macromol. 253 (Pt 7), 127467. 10.1016/j.ijbiomac.2023.127467 37863141

[B103] Pellerano M. Tcherniuk S. Perals C. Ngoc Van T. N. Garcin E. Mahuteau-Betzer F. (2017). Targeting conformational activation of CDK2 kinase. Biotechnol. J. 12 (8). 10.1002/biot.201600531 28430399

[B104] Petrova V. Arkhypov I. Weber R. Groth C. Altevogt P. Utikal J. (2020). Modern aspects of immunotherapy with checkpoint inhibitors in melanoma. Int. J. Mol. Sci. 21 (7). 10.3390/ijms21072367 PMC7178114 32235439

[B105] Peyregne V. P. Kar S. Ham S. W. Wang M. Wang Z. Carr B. I. (2005). Novel hydroxyl naphthoquinones with potent Cdc25 antagonizing and growth inhibitory properties. Mol. Cancer Ther. 4 (4), 595–602. 10.1158/1535-7163.Mct-04-0274 15827333

[B106] Plattner C. Lamberti G. Blattmann P. Kirchmair A. Rieder D. Loncova Z. (2023). Functional and spatial proteomics profiling reveals intra- and intercellular signaling crosstalk in colorectal cancer. iScience 26 (12), 108399. 10.1016/j.isci.2023.108399 38047086 PMC10692669

[B107] Reynolds R. A. Yem A. W. Wolfe C. L. Deibel M. R. Jr. Chidester C. G. Watenpaugh K. D. (1999). Crystal structure of the catalytic subunit of Cdc25B required for G2/M phase transition of the cell cycle. J. Mol. Biol. 293 (3), 559–568. 10.1006/jmbi.1999.3168 10543950

[B108] Rifaioglu A. S. Atas H. Martin M. J. Cetin-Atalay R. Atalay V. Doğan T. (2019). Recent applications of deep learning and machine intelligence on *in silico* drug discovery: methods, tools and databases. Brief. Bioinform 20 (5), 1878–1912. 10.1093/bib/bby061 30084866 PMC6917215

[B109] Rodon J. Soria J. C. Berger R. Miller W. H. Rubin E. Kugel A. (2019). Genomic and transcriptomic profiling expands precision cancer medicine: the WINTHER trial. Nat. Med. 25 (5), 751–758. 10.1038/s41591-019-0424-4 31011205 PMC6599610

[B110] Roelands J. van der Ploeg M. Ijsselsteijn M. E. Dang H. Boonstra J. J. Hardwick J. C. H. (2023). Transcriptomic and immunophenotypic profiling reveals molecular and immunological hallmarks of colorectal cancer tumourigenesis. Gut 72 (7), 1326–1339. 10.1136/gutjnl-2022-327608 36442992 PMC10314051

[B111] Rosario S. R. Long M. D. Affronti H. C. Rowsam A. M. Eng K. H. Smiraglia D. J. (2018). Pan-cancer analysis of transcriptional metabolic dysregulation using the Cancer Genome Atlas. Nat. Commun. 9 (1), 5330. 10.1038/s41467-018-07232-8 30552315 PMC6294258

[B112] Russell P. Nurse P. (1986). cdc25+ functions as an inducer in the mitotic control of fission yeast. Cell 45 (1), 145–153. 10.1016/0092-8674(86)90546-5 3955656

[B113] Salameh L. Bhamidimarri P. M. Saheb Sharif-Askari N. Dairi Y. Hammoudeh S. M. Mahdami A. (2022). Silico bioinformatics followed by molecular validation using archival FFPE tissue biopsies identifies a panel of transcripts associated with severe asthma and lung cancer. Cancers (Basel) 14 (7). 10.3390/cancers14071663 PMC8996975 35406434

[B114] Sarkis M. Miteva M. A. Dasso Lang M. C. Jaouen M. Sari M. A. Galcéra M. O. (2017). Insights into the interaction of high potency inhibitor IRC-083864 with phosphatase CDC25. Proteins 85 (4), 593–601. 10.1002/prot.25236 28056492

[B115] Schilsky R. L. (2010). Personalized medicine in oncology: the future is now. Nat. Rev. Drug Discov. 9 (5), 363–366. 10.1038/nrd3181 20431568

[B116] Schulz D. Wetzel M. Eichberger J. Piendl G. Brockhoff G. Wege A. K. (2021). Differential expression of PD-L1 during cell cycle progression of head and neck squamous cell carcinoma. Int. J. Mol. Sci. 22 (23). 10.3390/ijms222313087 PMC8658507 34884892

[B117] Semreen A. M. Alsoud L. O. El-Huneidi W. Ahmed M. Bustanji Y. Abu-Gharbieh E. (2022). Metabolomics analysis revealed significant metabolic changes in brain cancer cells treated with paclitaxel and/or etoposide. Int. J. Mol. Sci. 23 (22), 13940. 10.3390/ijms232213940 36430415 PMC9693830

[B118] Shao J. Ma J. Zhang Q. Li W. Wang C. (2023). Predicting gene mutation status via artificial intelligence technologies based on multimodal integration (MMI) to advance precision oncology. Semin. Cancer Biol. 91, 1–15. 10.1016/j.semcancer.2023.02.006 36801447

[B119] Shimazawa R. Suzuki T. Dodo K. Shirai R. (2004). Design and synthesis of dysidiolide analogs from vitamin D3: novel class of Cdc25A inhibitors. Bioorg Med. Chem. Lett. 14 (12), 3291–3294. 10.1016/j.bmcl.2004.03.100 15149692

[B120] Sicklick J. K. Kato S. Okamura R. Schwaederle M. Hahn M. E. Williams C. B. (2019). Molecular profiling of cancer patients enables personalized combination therapy: the I-PREDICT study. Nat. Med. 25 (5), 744–750. 10.1038/s41591-019-0407-5 31011206 PMC6553618

[B121] Sohn J. Kristjánsdóttir K. Safi A. Parker B. Kiburz B. Rudolph J. (2004). Remote hot spots mediate protein substrate recognition for the Cdc25 phosphatase. Proc. Natl. Acad. Sci. U. S. A. 101 (47), 16437–16441. 10.1073/pnas.0407663101 15534213 PMC534539

[B122] Srivastava S. Jayaswal N. Kumar S. Sharma P. K. Behl T. Khalid A. (2024). Unveiling the potential of proteomic and genetic signatures for precision therapeutics in lung cancer management. Cell Signal 113, 110932. 10.1016/j.cellsig.2023.110932 37866667

[B123] Stanford S. M. Bottini N. (2023). Targeting protein phosphatases in cancer immunotherapy and autoimmune disorders. Nat. Rev. Drug Discov. 22 (4), 273–294. 10.1038/s41573-022-00618-w 36693907 PMC9872771

[B124] Sur S. Agrawal D. K. (2016). Phosphatases and kinases regulating CDC25 activity in the cell cycle: clinical implications of CDC25 overexpression and potential treatment strategies. Mol. Cell Biochem. 416 (1-2), 33–46. 10.1007/s11010-016-2693-2 27038604 PMC4862931

[B125] Tang C. Fu S. Jin X. Li W. Xing F. Duan B. (2023). Personalized tumor combination therapy optimization using the single-cell transcriptome. Genome Med. 15 (1), 105. 10.1186/s13073-023-01256-6 38041202 PMC10691165

[B126] Tanoli Z. Vähä-Koskela M. Aittokallio T. (2021). Artificial intelligence, machine learning, and drug repurposing in cancer. Expert Opin. Drug Discov. 16 (9), 977–989. 10.1080/17460441.2021.1883585 33543671

[B127] Tao Y. Hao X. Jing L. Sun L. Cherukupalli S. Liu S. (2021). Discovery of potent and selective Cdc25 phosphatase inhibitors via rapid assembly and *in situ* screening of Quinonoid-focused libraries. Bioorg Chem. 115, 105254. 10.1016/j.bioorg.2021.105254 34426152

[B128] Tarawneh N. Hamadneh L. Abu-Irmaileh B. Shraideh Z. Bustanji Y. Abdalla S. (2023). Berberine inhibited growth and migration of human colon cancer cell lines by increasing phosphatase and tensin and inhibiting aquaporins 1, 3 and 5 expressions [article]. Molecules 28 (9), 3823. 10.3390/molecules28093823 37175233 PMC10180100

[B129] Trédan O. Wang Q. Pissaloux D. Cassier P. de la Fouchardière A. Fayette J. (2019). Molecular screening program to select molecular-based recommended therapies for metastatic cancer patients: analysis from the ProfiLER trial. Ann. Oncol. 30 (5), 757–765. 10.1093/annonc/mdz080 30865223

[B130] Tsimberidou A. M. Fountzilas E. Nikanjam M. Kurzrock R. (2020). Review of precision cancer medicine: evolution of the treatment paradigm. Cancer Treat. Rev. 86, 102019. 10.1016/j.ctrv.2020.102019 32251926 PMC7272286

[B131] Ugai T. Sasamoto N. Lee H. Y. Ando M. Song M. Tamimi R. M. (2022). Is early-onset cancer an emerging global epidemic? Current evidence and future implications. Nat. Rev. Clin. Oncol. 19 (10), 656–673. 10.1038/s41571-022-00672-8 36068272 PMC9509459

[B132] Vandekerkhove G. Struss W. J. Annala M. Kallio H. M. L. Khalaf D. Warner E. W. (2019). Circulating tumor DNA abundance and potential utility in *de novo* metastatic prostate cancer. Eur. Urol. 75 (4), 667–675. 10.1016/j.eururo.2018.12.042 30638634

[B133] Vartak R. Deore B. Sanhueza C. A. Patel K. (2023). Cetuximab-based PROteolysis targeting chimera for effectual downregulation of NSCLC with varied EGFR mutations. Int. J. Biol. Macromol., 252, 126413. 10.1016/j.ijbiomac.2023.126413 37598823 PMC12045033

[B134] Vigo E. Müller H. Prosperini E. Hateboer G. Cartwright P. Moroni M. C. (1999). CDC25A phosphatase is a target of E2F and is required for efficient E2F-induced S phase. Mol. Cell Biol. 19 (9), 6379–6395. 10.1128/mcb.19.9.6379 10454584 PMC84608

[B135] Viswanathan A. N. Erickson B. Gaffney D. K. Beriwal S. Bhatia S. K. Lee Burnett O. (2014). Comparison and consensus guidelines for delineation of clinical target volume for CT- and MR-based brachytherapy in locally advanced cervical cancer. Int. J. Radiat. Oncol. Biol. Phys. 90 (2), 320–328. 10.1016/j.ijrobp.2014.06.005 25304792 PMC4194192

[B136] Wang J. Li X. Chen S. Cao J. Fan X. Wang H. (2023). Identification of the role of MCM6 in bladder cancer prognosis, immunotherapy response, and *in vitro* experimental investigation using multi-omics analysis. Life Sci. 335, 122253. 10.1016/j.lfs.2023.122253 37951536

[B137] Wang J. Y. Yeh C. L. Chou H. C. Yang C. H. Fu Y. N. Chen Y. T. (2011). Vaccinia H1-related phosphatase is a phosphatase of ErbB receptors and is down-regulated in non-small cell lung cancer. J. Biol. Chem. 286 (12), 10177–10184. 10.1074/jbc.M110.163295 21262974 PMC3060470

[B138] Wang R. Dai W. Gong J. Huang M. Hu T. Li H. (2022). Development of a novel combined nomogram model integrating deep learning-pathomics, radiomics and immunoscore to predict postoperative outcome of colorectal cancer lung metastasis patients. J. Hematol. Oncol. 15 (1), 11. 10.1186/s13045-022-01225-3 35073937 PMC8785554

[B139] Wang Y. Zhao C. Chang L. Jia R. Liu R. Zhang Y. (2019). Circulating tumor DNA analyses predict progressive disease and indicate trastuzumab-resistant mechanism in advanced gastric cancer. EBioMedicine 43, 261–269. 10.1016/j.ebiom.2019.04.003 31031019 PMC6562020

[B140] Wegener S. Hampe W. Herrmann D. Schaller H. C. (2000). Alternative splicing in the regulatory region of the human phosphatases CDC25A and CDC25C. Eur. J. Cell Biol. 79 (11), 810–815. 10.1078/0171-9335-00115 11139144

[B141] Wekking D. Leoni V. P. Lambertini M. Dessì M. Pretta A. Cadoni A. (2023). CDK4/6 inhibition in hormone receptor-positive/HER2-negative breast cancer: biological and clinical aspects. Cytokine & Growth Factor Rev. 10.1016/j.cytogfr.2023.10.001 37838584

[B142] Wu Q. Yu J. Zhang M. Xiong Y. Zhu L. Wei B. (2024a). Serum lipidomic profiling for liver cancer screening using surface-assisted laser desorption ionization MS and machine learning. Talanta, 268, 125371. 10.1016/j.talanta.2023.125371 37931569

[B143] Wu Y. Wu M. Zheng X. Yu H. Mao X. Jin Y. (2024b). Discovery of a potent and selective PARP1 degrader promoting cell cycle arrest via intercepting CDC25C-CDK1 axis for treating triple-negative breast cancer. Bioorg Chem. 142, 106952. 10.1016/j.bioorg.2023.106952 37952486

[B144] Xiao Y. Yu Y. Gao D. Jin W. Jiang P. Li Y. (2019). Inhibition of CDC25B with WG-391d impedes the tumorigenesis of ovarian cancer. Front. Oncol. 9, 236. 10.3389/fonc.2019.00236 31024841 PMC6463794

[B145] Xu X. Yamamoto H. Liu G. Ito Y. Ngan C. Y. Kondo M. (2008). CDC25A inhibition suppresses the growth and invasion of human hepatocellular carcinoma cells. Int. J. Mol. Med. 21 (2), 145–152. 10.3892/ijmm.21.2.145 18204780

[B146] Xu X. Yamamoto H. Sakon M. Yasui M. Ngan C. Y. Fukunaga H. (2003). Overexpression of CDC25A phosphatase is associated with hypergrowth activity and poor prognosis of human hepatocellular carcinomas. Clin. Cancer Res. 9 (5), 1764–1772. 12738732

[B147] Yoshitome S. Aiba Y. Yuge M. Furuno N. Watanabe M. Nakajo N. (2019). Involvement of Myt1 kinase in the G2 phase of the first cell cycle in *Xenopus laevis* . Biochem. Biophysical Res. Commun., 515(1), 139–144. 10.1016/j.bbrc.2019.05.104 31128913

[B148] Yu N. Hwang M. Lee Y. Song B. R. Kang E. H. Sim H. (2023). Patient-derived cell-based pharmacogenomic assessment to unveil underlying resistance mechanisms and novel therapeutics for advanced lung cancer. J. Exp. Clin. Cancer Res. 42 (1), 37. 10.1186/s13046-023-02606-3 36717865 PMC9885631

[B149] Zeggini E. Gloyn A. L. Barton A. C. Wain L. V. (2019). Translational genomics and precision medicine: moving from the lab to the clinic. Science 365 (6460), 1409–1413. 10.1126/science.aax4588 31604268

[B150] Zhang S. Gao Q. Li W. Zhu L. Shang Q. Feng S. (2019). Shikonin inhibits cancer cell cycling by targeting Cdc25s. BMC Cancer 19 (1), 20. 10.1186/s12885-018-5220-x 30616572 PMC6323793

[B151] Zhao S. Li Y. Li G. Ye J. Wang R. Zhang X. (2023). PI3K/mTOR inhibitor VS-5584 combined with PLK1 inhibitor exhibits synergistic anti-cancer effects on non-small cell lung cancer. Eur. J. Pharmacol., 957, 176004. 10.1016/j.ejphar.2023.176004 37625683

